# Object Recognition at Higher Regions of the Ventral Visual Stream via Dynamic Inference

**DOI:** 10.3389/fncom.2020.00046

**Published:** 2020-06-23

**Authors:** Siamak K. Sorooshyari, Huanjie Sheng, H. Vincent Poor

**Affiliations:** ^1^Department of Integrative Biology, University of California, Berkeley, Berkeley, CA, United States; ^2^Department of Electrical Engineering, Princeton University, Princeton, NJ, United States

**Keywords:** object recognition, sequence estimation, decoding, IT cortex, dynamic inference, Viterbi algorithm

## Abstract

The ventral visual stream (VVS) is a fundamental pathway involved in visual object identification and recognition. In this work, we present a hypothesis of a sequence of computations performed by the VVS during object recognition. The operations performed by the inferior temporal (IT) cortex are represented as not being akin to a neural-network, but rather in-line with a dynamic inference instantiation of the untangling notion. The presentation draws upon a technique for dynamic maximum a posteriori probability (MAP) sequence estimation based on the Viterbi algorithm. Simulation results are presented to show that the decoding portion of the architecture that is associated with the IT can effectively untangle object identity when presented with synthetic data. More importantly, we take a step forward in visual neuroscience by presenting a framework for an inference-based approach that is biologically inspired via attributes implicated in primate object recognition. The analysis will provide insight in explaining the exceptional proficiency of the VVS.

## 1. Introduction

A prevalent hypothesis is that the identities of viewed objects are represented as patterns of activity across populations of neurons with increasingly complicated computations occurring further along the ventral visual stream (VVS). Presenting a biologically inspired algorithm where the stimulus information is processed and exchanged among different populations of neurons is a challenge. Since the higher visual areas such as inferior temporal (IT) cortex are selective to the more complex stimuli characteristics than populations in lower levels such as V1 and V2, it has been postulated that more complicated processing techniques are used by the IT (Riesenhuber and Poggio, 1999). The term “encoding” has been applied extensively to the manner by which neurons in the early visual stages respond to and represent stimuli. The presented analysis will treat the object recognition process performed by the higher regions of the VVS as a decoding operation and present a model that can commence to unify an understanding of the computations involved during such a cognitive process. Topical overviews such as DiCarlo et al. (2012) advocate the first step of unequivocally defining the question of how the brain solves the problem. It is sensible to presume that as large amounts of data become available the object recognition question will be asked in different ways. Computer vision algorithms have been lauded for efficacy in categorizing objects after being trained on large sets of sample data. However, they are also known to suffer from the invariance problem that has been studied by visual neuroscientists. This is especially true when a large number of object categories are considered, and imparts one to question whether computer vision models are the optimal means for studying the computational operations performed by the brain during real-time object recognition. The encoding-decoding methodology discussed in this work will provide a model that is a closer, biologically-plausible explanation of the VVS operation.

Neurons at progressive stages of neuroanatomy receiving weighted excitatory and inhibitory inputs prior to their state being subject to a thresholding operation is not a new concept to vision neuroscience. We consider somewhat more sophisticated operations that will occur over several populations of neurons. While the algorithmic operations may be deemed sophisticated, it is noteworthy that such operations are being performed by millions of neurons. Furthermore, the fact that primates are extremely efficient in conducting object recognition vindicates the use of algorithms to explain the seemingly effortless manner by which the recognition is performed. The input to the model will be the representation that the viewed object should evoke at the IT. This representation is obviously associated with the visual stimulus, and is immediately encoded by the retina and lateral geniculate nucleus (LGN) circuitry in order for its meaning to be communicated along the VVS in a reliable manner. The model presented in this work provides an alternative to neural network techniques employing max-pooling, and an alternative to machine learning approaches that consider object categorization rather than classification of object attributes during the recognition process. The analysis additionally brings forth the question of what metrics to consider in assessing how well a model performs object recognition. Within the encoding-decoding framework it is possible to distinguish between different gradations of recognition. Specifically, one would be able to quantify the error rate in recognizing objects, the attributes of an object, and the object category.

**Figure 1:**
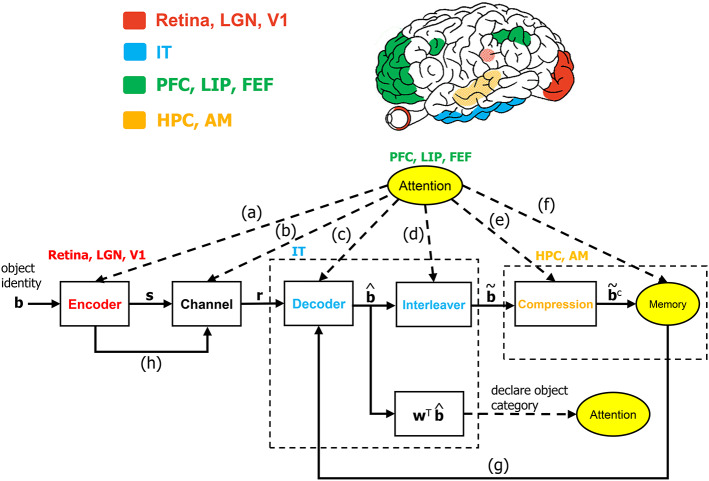
The communication-theoretic object recognition (CTOR) model. The structure of CTOR consists of feedforward processing with memory and attention providing feedback to the decoder portion of the IT. **(a)** The communication of *T*, {α_*i*_}, and {*G*_*i*_(*t*)} of equations (1) and (2) from the PFC, LIP, and FEF regions to V1, V2, and V4. **(b)** Attention modulating the channel properties. **(c,g)** The communication of the transition probabilities {*P*[**r**_*k*_|**b**_*k*+1_, **b**_*k*_]} and the priors {*P*[**b**_*k*+1_|**b**_*k*_]} to the IT from the attention and memory circuitry, respectively. **(d)** The conveying of the number of features *F* and the number of bits allocated to each feature {*M*_*i*_} by attention to the interleaver. **(e)** Conveyance of the degree of compression from attention. **(f)** Attention gating memory as far as the object features that are retained following recognition. **(h)** The contribution of the retinal, LGN, and V1 stages to the neural noise process constituting the channel. The corresponding brain regions are marked in the brain above the diagram. The units drawn with solid lines are modeled by algorithmic operations. Filled areas represent brain regions on the surface, while shaded areas represent those embedded inside the brain. LGN, lateral geniculate nucleus; V1, primary visual cortex; IT, inferior temporal cortex; LIP, lateral intraparietal area; FEF, frontal eye fields; HPC, hippocampus; AM, amygdala.

Algorithmic operations will be suggested herein for various stages of the VVS to mirror the functional operations implicated by prior works in visual neuroscience. The algorithmic structure in Figure 1 is novel within the context of visual neuroscience. The biologically-inspired system will be referred to as the communication-theoretic object recognition (CTOR) model and will encompass high-level visual function processing low-level sensory signals. A natural impetus for the derivation of CTOR is the brain consisting of communication channels with a task such as object recognition invoking the interchange of signals between neural circuits as part of the interplay between top-down and bottom-up processing. There are several themes that subsist when considering statistical inference on the output of a non-ideal channel in engineering or biology: the time-sensitive nature of the information, the presence of stochastic perturbations, and the possible compression of the recovered information. Refinements of CTOR that may spawn from this presentation will need to include a decoding algorithm for inference. Indeed, alternate decoding algorithms may be proposed and different definitions for the elements that comprise the decoded sequence may emerge.

## 2. Attributes of Bio-inspired Object Recognition Models

It has been reputed that neural connectivity dictates a hierarchical organization at the VVS with visual information traversing the retina to the LGN, and then through cortical area V1, V2, and V4 before reaching the IT. Neurons in V1 have small receptive fields and respond to simple features such as edge orientation (Hubel and Wiesel, 1962). The receptive fields of V4 neurons are on average four to seven times greater than those in V1, but are smaller than the receptive fields of IT neurons. Many V4 neurons are sensitive to stimulus features of moderate complexity (Cadieu et al., 2007), whereas the IT neurons are selective to much more complex stimuli such as faces. The tuning properties of IT cells seem to be shaped by task learning with their dendritic arbors being more expansive than those of V1, V2, or V4 neurons (Elston, 2002; Luebke, 2017). The untangling notion advocated in DiCarlo et al. (2012) serves as motivation for the decoding module in CTOR. As the viewed object is processed beyond the retina and along the successive stages of the VVS, it is believed that increasingly sophisticated processing power is applied to untangle the object's identity. When considering the statistics of the input to the lowest stage in the model, works such as Simoncelli and Olshausen (2001) have provided a litany of studies that contain empirical evidence for the non-Gaussianity of natural images. The authors proceed to describe the neural coding/representation that occurs in portions of the visual cortex. An array of works have discussed attributes of the visual cortex that enable the system to be exceptionally proficient at performing object recognition in a rapid and effortless manner. The following are what we consider the most crucial attributes that a biologically-inspired model for object recognition should address.

- *Selectivity*: The ability to accurately discriminate between different objects. Object recognition models typically do not quantitatively distinguish between object identification and categorization. The model herein will distinguish between the two domains and focus on the identification of an object rather than a rapid categorization.
- *Invariance*: The ability to recognize an object under transformations such as scale or position alterations in the field of view. Furthermore, inconsiderable alternations in the object's features should not preclude recognition.
- *Robustness*: Aspects of the viewed stimulus such as illumination and clutter may decrease the signal-to-noise ratio (SNR) of the neural signals communicated along the lower visual stages. The VVS is frequently able to distinguish among objects in light of perturbations to the viewed object that reduce the SNR of the neural information progressing along the pathway.
- *Processing Speed*: The recognition of an object within either a strict or lax temporal constraint imposed by the task. From a psychophysics perspective, the processing speed corresponds to how rapidly the object recognition is performed by the brain.
- *Attentional Gating*: The degree and implications of attention allocated to recognizing an object. The dynamics of the allocated attention will govern how the brain parses object features and what is retained following recognition.
- *Dynamic Recurrence*: The consideration of feedback as a necessary complement to the feedforward processing. The recurrence should be dynamic and involve interaction between multiple brain areas.

The first two attributes have been discussed in works such as Serre and Riesenhuber (2004), whereas robustness has been considered in a multitude of studies (e.g., Cadieu et al., 2007). The processing speed was elegantly discussed in Thorpe and Van Rullen (2001), while attentional modulation during object recognition has also been extensively investigated in the literature. The processing speed and attentional gating attributes will have analogues in CTOR. Dynamic recurrence in the VVS during object recognition has been experimentally instantiated by works such as Wyatte et al. (2012), O'Reilly et al. (2013), and Poggio and Kreiman (2013). It seems natural for the brain to take advantage of feedback pathways to coordinate between top-down and bottom-up signals during more challenging recognition tasks such as object completion or identification in the presence of clutter. In fact, studies on neural circuit specialization and connectivity have discussed areas V1 and V2 receiving connections from IT and parahippocampal regions (Rockland, 1997). Consideration of the above attributes presents an avenue to discuss how CTOR is a bio-inspired model for object recognition at the VVS. Primate circuits such as the cerebral cortex, hippocampus, and amygdala are associated with advanced cognitive functions and have been shown to contain pyramidal neurons whose architecture seem to be specialized for the posited task of such neural circuits (Jacobs and Scheibel, 2002; Elston, 2003). Interestingly, substantial differences are noted in the number of spines on the basal dendritic fields of neurons in V1, V2, and IT with the quantity and density multiplicatively increasing when progressing from V1 to IT. This is believed to lead to the increased capability of pyramidal neurons in the latter stages of information processing such as the IT and PFC to integrate a broader range of synaptic inputs than neurons at the lower cortical areas such as V1 and V2 (Elston et al., 1999). Thus, the anatomy and connectivity of the cortical circuitry are crucial in determining any prospective computation (Elston, 2003; Spruston, 2008; Luebke, 2017). The intriguing discussion of Biederman (1987) brought forth the recognition-by-components (RBC) view of vision where it was suggested that the brain parses viewed objects into parts. Partial matches among the segments are then possible, and the proportion of the similarity in the components between the viewed object and a stored representation is used to assess the fidelity of the match. Elements of CTOR have been motivated by the valuable discussion in (Biederman, 1987) and the presented model aims to further concretize RBC.

## 3. Modeling Object Recognition as Dynamic Inference

It is reputed that a study of how the neural populations of the visual system process scenes so that the brain is capable of object recognition leads to an overcomplete problem. In a nominal example an information-rich scene is presented to a subject with an object of interest embedded in the scene. Regardless of the object's salience, the subject has been provided with a plethora of visual information for the prospective task. The hierarchical and non-linear nature of the layers that govern the computations among simple and cortical complex cells implicate the difficulty of formulating optimization functions that the visual system may be attempting to minimize/maximize during such a nominal task. It has also been argued that the difficulty in attempting to mimic functions of the visual cortex is further complicated by its columnar organization and the heterogeneity among the columns Roe (2019). In light of this, works such as Serre et al. (2007) have motivated the approach of studying each layer in the system separately. We believe that a graceful unison should exist between the two disparate avenues of viewing the system as a whole and dividing it into disjoint units. Figure 1 depicts the architecture that will be motivated as a sensible hypothesis for high-level computational processing occurring in the VVS during object recognition. The conjecture is unique since it is biologically inspired to reflect the VVS's operation while concomitantly being an ideology borrowed from communication theory. From a communication-theoretic perspective, the seminal work of Shannon (1948) has led to countless developments in the design of structured redundancy applied to information that is conveyed over a noisy channel to a receiver with processing capability. The transmission of such structured redundancy is often perturbed in a stochastic manner by a channel prior to it being decoded, or more appropriately for this presentation, “untangled” by the destination. The necessary background on the encoder-channel-decoder structure within a communication-theoretic setting has been provided in Fano (1963) as well as classical texts such as Wozencraft and Jacobs (1965).

**Figure 2:**
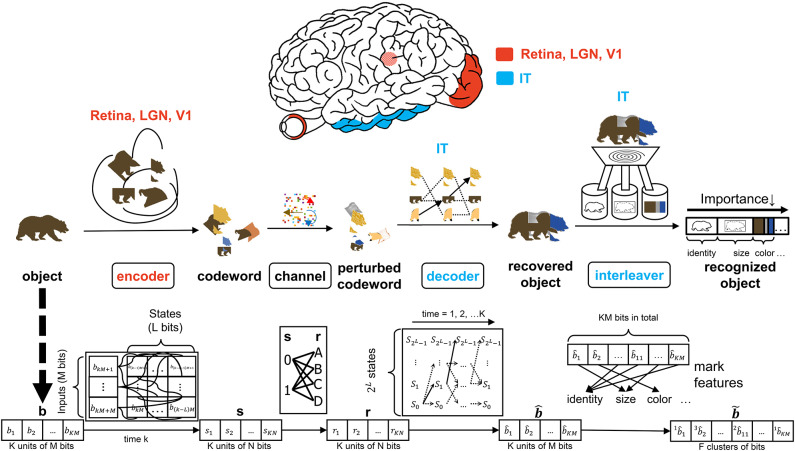
The interaction of the input, encoder, channel, decoder, and interleaver in the CTOR model. The top row depicts the brain regions implicated for the encoding, decoding, and interleaving operations. The middle row provides an illustration of CTOR operations with the viewed object being a brown bear. The bottom row illustrates the progression of the dimensions of the neural signals that are subject to the CTOR operations.

It is evident that psychological processes such as attention and memory are prerequisites for visual perception. There is a wealth of literature on the computational capacity of cortical circuitry and the quantitative differences among the population of neurons associated with vision—see Elston, 2002; Jacobs and Scheibel, 2002; Spruston, 2008; Elston and Fujita, 2014; Luebke, 2017 for reviews. The work of Mishkin has provided clear evidence for the inclusion of the hippocampus and amygdala in the so-called recognition memory circuitry. In fact, Mishkin (1982) concludes that a model of object recognition would be incomplete without considering recognition memory and the corresponding feedback and feedforward projections to the hippocampus and amygdala. Furthermore, the pyramidal neurons present in the visual cortex are also seen in the hippocampus and the amygdala (Feldman, 1984; DeFelipe and Farinas, 1992). The notion of re-integration is also advocated by Mishkin; lending credence to the presence of concatenated operations such as the decoder in Figure 1 being followed by an interleaving operation. The hierarchical nature of the visual system consists of bi-directional information flow between the various levels (Van Essen and Gallant, 1994). Studies such as O'Reilly et al. (2013) and Lamme and Roelfsema (2000) have advocated the interaction of feedforward and feedback processing in delineating between the quick and detailed categorization of an object. The architecture of Figure 2 considers feedforward connections as well as feedback projections that are guided by memory and the neural circuitry associated with attention. It is noteworthy that neuroanatomical evidence for cell structure influencing function in the visual system is provided in studies such as Elston et al. (2005) and Jacobs et al. (2001), and there are abundant discussions on the specialization of feedforward and feedback connections along the VVS (Rockland, 1997).

The CTOR formulation is fundamentally different from prior computational vision works such as Salinas and Abbott (1997) by considering the operation of the IT neural circuitry along with the functionality posited to be performed by the lower layers of the visual system. The notion that visual objects are represented by patterns of activity across populations of neurons has been advocated in discussions such as Zhang et al. (2011) and Lee and Mumford (2003). In accordance, the processing considered in CTOR can incorporate the representation of the neural activity via vectors that have dimensionality corresponding to the considered neural population. The encoding and decoding operations hypothesize that the neural activity has structure and is affected by the external environment and a subject's memory. Attention is suggested as having an impact on all operations of CTOR including the encoder, channel, decoder, interleaver, compression, and memory (Figure 1). The work of Lee and Mumford (2003) presented Bayesian inference as part of a graphical model for the viewing of an object by the early visual cortex. Their analysis makes mention of neural populations from the IT and V4, but is primarily focused on V1 and V2. The CTOR formulation will focus on the higher visual regions by presenting maximum a posteriori probability (MAP) inference within the context of the IT's role in object recognition. We shall use a binary alphabet to present the signals at the various stages of CTOR, however, the components should not be automatically associated with spikes. It is logical to inquire if the elements of the encoded and decoded CTOR signals are outputs of individual neurons, the result of a principal component analysis applied to output of populations of neurons, or perhaps the binary-thresholded outputs of neural circuits. The dimensionality of the signals in Figure 1 can be specified to encompass all of the aforementioned scenarios. While such level of abstraction may be deemed unnecessary, it is productive for a new model to allow flexibility so that it can be fit to various data sets. As advocated in works such as DiCarlo and Cox (2007), CTOR encourages a shift in emphasis from single-unit spiking activity in favor of the processing performed by neural circuits.

### 3.1. Model Input

A seemingly fundamental facet of a model is the input. Object *i* will be denoted by a binary representation **b**^*i*^ that encompasses the object's attributes. The stimulus index *i* = 1, 2, 3, … will serve as the identity of the viewed object and the representation that the object should evoke at the IT for correct recognition. The representation **b** will be tangled by the retina, LGN, and V1 prior to being untangled by the IT. In a nominal object recognition trial the stimulus representation of a viewed object such as a brown bear (Figure 2) will be faithfully recovered by the IT and then compressed prior to being stored in memory. The tangling of the object identity prior to its progression along the VVS has been elegantly discussed in DiCarlo and Cox (2007) via the notion of an intertwining of object manifolds. CTOR provides a concrete means of representing such a tangling, namely the mapping of **b** to a codeword as will be discussed below.

### 3.2. Object Tangling via the Encoder

The early stages of the visual system will tangle the representation **b** that the viewed stimulus should evoke at the IT. The CTOR example illustrated in Figure 2 considers the encoder as being stimulus-driven. A rate coding operation has been advocated as taking place in various visual areas (Van Essen and Gallant, 1994). The viewed object manifolds conveyed to area V1 by the retinal and LGN processing are nearly as tangled as the pixel representation (DiCarlo et al., 2012). This is largely attributed to the receptive fields in the aforementioned two populations being functionally akin to point-wise spatial filters (Olshausen and Field, 2005). Interestingly, as the retinal- and LGN-processed signals are processed by V1, the total dimensionality of the representation is increased approximately 30-fold (Stevens, 2001). However, the V1-processed signal is still considered highly tangled since its response is significantly inferior to human performance for real-world recognition problems (DiCarlo et al., 2012). Such biological characteristics are motivation for CTOR to postulate the encoder as being comprised of the retina, LGN, and V1 circuitry. Since the object representation is tangled by the encoder, it is debatable whether LGN—rather than V1—should be considered as the last stage of the encoder. This judgment is based on the V1 output still being highly tangled, and that the dimensionality increase that occurs following V1's processing of the LGN output is a trademark of the encoding operation. An example is shown in Figure 2 where the representation **b** of a viewed object, i.e., a brown bear, is encoded into the stream **s** as the tangled version of the representation which should be evoked at the IT when viewing this object. Two parameters are crucial to the discussion. Assuming a binary alphabet, the integer *M* will denote the number of input bits processed by the encoding stage at a time. The integer *K* will denote the number of *M*-bit units allocated for representing the viewed object. Thus, the IT representation of a viewed object will consist of *KM* bits, and the IT may have 2^*KM*^ distinct representations for a temporal window of duration *K*. The CTOR model considers a continuous stream of input bits being processed by the VVS. The continuous stream of information has been segmented into *KM* bits at different object boundaries. In effect, a larger *K* will correspond to an increase in the complexity of the viewed object.

Anatomically, the output of the encoder circuitry will be a length *KN* codeword **s** that comprises the neuronal response that the IT must decode (Figure 2). Although exceptionally large, we shall consider the number of possible representations as being finite. From a communication-theoretic perspective, encoding is an operation where a *M*-component input is mapped to a message consisting of *N* ≥ *M* components. From a reliability perspective it is advantageous to have *N*≫*M* because it behooves the decoder to have access to as many information-bearing signals as possible in its decision of which message to declare as the untangled representation. The ratio *M*/*N* ≤ 1 is dubbed the code rate and the *N* = *M* scenario is the somewhat anomalous case referred to as rateless coding because it provides no redundancy. An important parameter stems from the non-restrictive assumption that the encoder generates the codewords via a shift-register structure (Lin and Costello, 1983). The maximal memory order of the shift register will be designated by *L*. For ease in presentation, we shall assume a simple shift-register structure where the total memory is equal to the maximal memory (*L*). In communication theory, this quantity is referred to as the encoder constraint length and the same name will be used henceforth. It shall be assumed that only one bit is fed into the encoder at each time instant (i.e., *M* = 1)—this is also a non-restrictive assumption that is made for ease of presentation. At each time instant there will be 2^*L*^ possible states $\left\{S_{0},S_{1},\ldots ,S_{2^{L}-1}\right\}$, that the encoder can take, and we shall denote the encoder state at time *k* by $S_{k,i}:i=0,1,\ldots ,2^{L}-1$. The time index *k* = 1, 2, 3, … will be suppressed unless when necessary. When in state *S*_*k,i*_ an encoder can produce only one of two possible codewords at time *k* + 1. Similarly, a generated codeword could have only been preceded by two possible codewords at time *k* − 1. The length-*N* codeword *s*_1_, *s*_2_, …, *s*_*N*_ at time *k* will be denoted by the vector **s**_*k*_. The *N* components of the output codeword **s**_*k*_ will be dependent on ${\text{b}}_{k}^{i}$ as well as the *L* prior inputs to the encoder: ${\text{b}}_{k-1}^{i},{\text{b}}_{k-2}^{i},\ldots ,{\text{b}}_{k-L}^{i}$. The codewords **s**_1_, **s**_2_, …, **s**_*K*_ for duration *K* are concatenated as the encoder output vector **s** (Figure 2). In evaluating the CTOR operation and performance in the ensuing sections we shall consider ${\text{b}}_{k}^{i}$ as being comprised of a small number of bits of synthetic data. The use of such synthetic data is a logical first step for introducing and motivating the model. In subsequent works an image stimuli can be considered by devising the sequences ${\text{b}}_{k-1}^{i},{\text{b}}_{k-2}^{i},\ldots ,{\text{b}}_{k-L}^{i}$ to be binary representations of the pixels in the object that is viewed by a subject. Expanding CTOR functionality to operate on input consisting of pixel intensities is a future consideration. As a summary, the biological implication of the encoder is relatively simple - a viewed object should elicit a representation at the IT; the representation is tangled via the encoding operation performed by the lower layers of the visual system. Assuming a binary alphabet, the neural signal corresponding to the encoded object will be represented by *KN* bits.

It is believed that the spiking of visual neurons is greater when attention is allocated to a stimulus than when attention has not been allocated to the same stimulus. The spiking rate of the retinal and V1 populations of neurons will be represented via the relation

(1) $$\begin{array}{l} s_{i}\left(t\right)=G_{i}\left(t\right)s_{i,\text{rest}}\left(t\right)\text{ for }i=1,2,\ldots N. \end{array}$$

**Figure 3:**
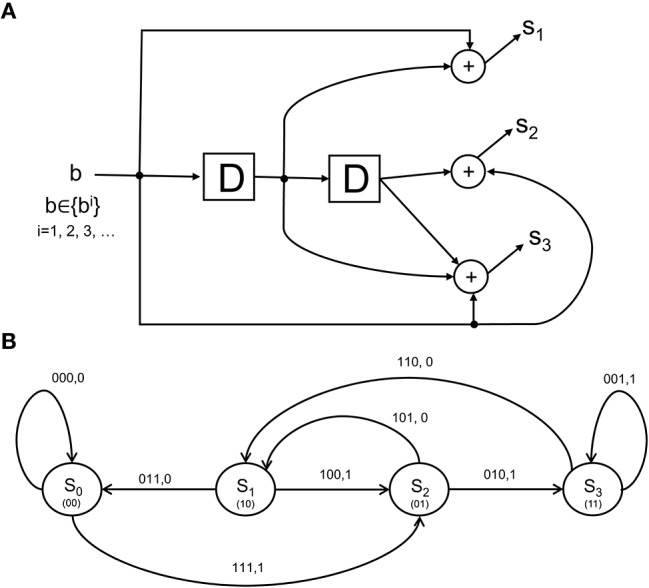
The structure and state diagram of a simple encoder. **(A)** The so-called simple-encoder considered as performing the tangling operation at the retina and LGN in Figure 1. The “D” elements denote a unit delay inherent in the encoder's processing. The above is a definite simplification of encoding performed by the neural circuitry, but will serve to illustrate an instance of CTOR's encoding operation when the early visual stages are presented with an object. **(B)** A state diagram representation depicting the dynamics of the encoder in **(A)**. The grouping 100, 1 above the state transition *S*_1_ → *S*_2_ indicates that when the neural circuits representing the retinal and V1 stages (i.e., the encoder) are in a state of *S*_1_, a stimulus value of 1 would result in a transition to state *S*_2_ as well as the encoder output of 100. The contents of the “D” elements in **(A)** representing the corresponding state are given between parentheses in **(B)**.

The above reflects attention, modeled by a positive and time-varying quantity *G*_*i*_(*t*) that has a multiplicative effect on the firing rate of the neurons. The process *s*_*i*,rest_(*t*) denotes the unmodulated firing rate of the *i*th V1 neuron. Works such as McAdams and Maunsell (1999) and Salinas and Abbott (1997) have provided evidence for *G*_*i*_(*t*) being a Gaussian function with parameters dependent on the attended location and the preferred attentional locus of the *i*th neuron. A codeword of length *N* denoted by a stream *s*_1_, *s*_2_, …, *s*_*N*_ will designate the activity of the V1 population of neurons, with the *i*th codeword component being “1” if the *i*th neuron has fired more than α_*i*_ > 0 times during an interval (e.g., 50 ms as noted in DiCarlo et al., 2012), and “0” otherwise. In other words, it is conceivable to consider an assignment

(2) $$s_{i}=\{\begin{array}{ll} 1 & \text{if }\int_{0}^{T}s_{i}\left(t\right)dt>\alpha_{i}\\ 0 & \text{otherwise} \end{array}$$

as the rate coding rule for each of the *N* units over a time epoch of *T* seconds. More elaborate scenarios can be concocted where sub-populations of the lower-visual level neurons each form codewords that are multiplexed to form a larger codeword that is signaled to the IT. The CTOR model will specify the codeword *s*_1_, *s*_2_, …, *s*_*N*_ constructed by an encoder with a state-machine structure such as that shown in Figure 3A. The state diagram in Figure 3B illustrates the input-output dynamics of this encoder, where it is evident from Figure 3A that the encoder output will depend on the prior inputs to the encoder. Biologically, this implies that the output of the retina, LGN, and V1 stage is not a memoryless sequence, but rather follows a pattern that is modulated by various processes.

### 3.3. The Channel

Visual recognition is affected by dynamic perturbations that can have impeding effects such as obfuscating the object identity, delaying the recognition, and possibly leading to an erroneous identification of the object or its characteristics. The hindrances might stem from the properties of the viewed object (e.g., the novelty, or the object being obscured in the scene), or a subject's attentional state. Since neurons are inherently noisy, it is also possible for the encoder to be imperfect during its encoding of the stimulus. The CTOR model will subsume such impediments within a channel that separates the encoding and decoding operations (Figure 2). The output of the channel will be denoted by the vector **r** and shall constitute the input to the IT. For clarity in presentation, and in cadence with the communication and information theory literature, we separate the encoder and channel in Figure 2 despite the fact that they are compound entities within a VVS. Although the channel separates the early visual stages from the IT, the early stages' operations will resonate in shaping the stochastic perturbations that are modeled via the channel.

The instantiation of a channel plays a role in studying the robustness attribute that we have discussed for object recognition. A channel provides a source of dispersion (Figure 2) by distorting the codeword and will be represented via a conditional distribution *P*[**r**|**s**] where **r** is a perturbed version of the signal and **s** is the encoder output. The channel may perturb the encoder output in either a continuous or discrete (i.e., quantized) fashion, accordingly, *P*[**r**|**s**] will be represented either by a probability density function (pdf) or a probability mass function (pmf), respectively. The simplest linear, continuous channel consists of a noise process **n** being added to the encoder output via

(3) $$\begin{array}{l} \text{r}=\text{s}+\text{n}. \end{array}$$

A prevalent channel quality metric (CQM) for a continuous channel is the SNR. For (3) the SNR of neural signals conveyed to the IT will be expressed as

(4) $$\begin{array}{l} {\text{SNR}}_{i}=\frac{\max\left(s_{i}\right)-\min\left(s_{i}\right)}{E\left[n_{i}^{2}\right]}=\frac{1}{E\left[n_{i}^{2}\right]}\text{ for }i=1,2,\ldots ,N \end{array}$$

with the denominator representing the neuronal noise power. The SNR of single neurons has been considered in numerous studies. In the spirit of works such as Mar et al. (1999), we consider an aggregate, population-wide CQM for the collective effect of the units comprising the retina, LGN, V1, V2, and V4. An insightful CQM for a discrete channel will quantify the uncertainty in the probabilistic mapping of the channel inputs to the channel outputs. The conditional entropy

(5) $$\begin{array}{r} H\left(r_{i}|s_{i}=n\right)=-\overset{\left|r\right|}{\underset{m=1}{\sum }}P\left[r_{i}=m|s_{i}=n\right]\log\left(P\left[r_{i}=m|s_{i}=n\right]\right)\\ \text{ for }n=0,1\text{ and }i=1,2,\ldots ,N \end{array}$$

is viewed as the equivocation between a discrete channel's input and output, with |*r*| denoting the cardinality of the set of possible channel outputs. From a biological perspective it is sensible to assume that over a short time-scale associated with a task, a continuous channel will maintain a probability distribution, but the parameters that characterize the distribution (e.g., mean and variance) will vary. Similarly, for a discrete channel it would be expected that during the viewing of an object the components of *P*[**r**|**s**] change but the values {*H*(*r*_*i*_|*s*_*i*_)} do not drastically vary. Over longer time-scales that span the viewing of different scenes it is expected that the channel's distribution will vary due to different stimuli and changes in attention.

### 3.4. The Decoder

There is evidence that in the visual cortex, neurons such as pyramidal cells become increasingly large, more branched, and more spinous as one progresses along the VVS (Elston, 2002). From the perspective of information transmission, the identity of a viewed object propagates along the VVS until reaching the IT. Works such as Karklin and Lewicki (2009) have suggested that sensory signals from early visual areas convey information that allows the higher visual areas to construct more complex representations of the sensory input. With CTOR, it is the objective of the decoder to determine the object identity and classify its characteristics. In effect, the decoded message will represent the object that the IT has identified from the representation propagated to the IT by the lower visual stages. After *K* time instances the sequence of vectors **r**_1_, **r**_2_, …, **r**_*K*_ will be available to the decoder with **r**_*k*_ representing a length-*N* perturbed codeword that is to be untangled into a length-*M* message. Accordingly, the decoder will continuously process the channel output at every time instant, with its output being a length-*KM* binary vector denoted by $\hat{\text{b}}$. The selectivity attribute discussed in section 2 is accounted for by the fact that the objective of decoding is discriminating between different patterns. A good decoder operating over a channel that is not inordinately dispersive will be capable of discriminating among various object representations with high likelihood. In effect, at each discrete time instant the decoder transforms a *N*-length sequence that may take on a number of possibilities to a *M*-length binary sequence. A decoding operation is conceived by considering various metrics, for example, a MAP decoder would select $\hat{\text{b}}={\text{b}}^{i}$ for the **b**^*i*^ that maximizes the probability *P*[**b**^*i*^|**r**] where *i* ∈ {1, 2, …, 2^*KM*^}. Dynamic programming is often used to solve large-scale inference problems when it is desired to recover a sequence that has the highest possibility of having occurred. The Viterbi algorithm provides the most probable sequence of states when the environment is described by a hidden Markov model (HMM) (Eddy, 2004) with the similarities between the principle and dynamic programming discussed in the seminal work of Forney (1973). A description of the Viterbi algorithm is provided in Appendix A and the terminology there will be incorporated henceforth. The CTOR proposal for the VVS proficiency at object recognition lies in the IT implementing the untangling notion via a MAP decoding algorithm in order to infer the object identity and attributes. The untangling notion can be equated to seeking the most likely path in a state transition diagram with 2^*L*^ states at time *k*. The length of prospective transitions between two states **b**_*k*+1_ and **b**_*k*_ at time *k* is quantified via

(6) $$\begin{array}{l} \lambda \left({\text{b}}_{k+1},{\text{b}}_{k}\right)=-\ln\left(P\left[{\text{b}}_{k+1}|{\text{b}}_{k}\right]\right)-\ln\left(P\left[{\text{r}}_{k}|{\text{b}}_{k+1},{\text{b}}_{k}\right]\right) \end{array}$$

where *P*[**b**_*k*+1_|**b**_*k*_] is the a priori probability of state **b**_*k*+1_ given the observance of state **b**_*k*_, and the transition probability *P*[**r**_*k*_|**b**_*k*+1_, **b**_*k*_] denotes the probability between a given pair of successive states and the sequence **r**_*k*_. The process is illustrated in the decoder portion of the example in Figure 2 with the IT performing dynamic sequence estimation of the tangled representation.

An appeasing feature of the CTOR proposal is that the invariance, robustness, and selectivity attributes discussed in section 2 may be considered in unison. This is because when decoding **r** the MAP sequence estimation technique attempts to recover the correct message, or one that is as “close” as possible to the correct message despite disparity in certain attributes. The disparity is noted by the bit streams disagreeing at various positions, and the degree of closeness is quantified by the Hamming distance between the sequence decoded by the IT and the representation that the viewed object should have evoked at the IT. We define the deviation by

(7) $$\begin{array}{l} d\left(\hat{\text{b}},{\text{b}}^{i}\right)≜{\left\|\hat{\text{b}}⊕{\text{b}}^{i}\right\|}_{0} \end{array}$$

where ⊕ denotes the component-wise XOR operation and ||**x**||_0_ denotes the number of non-zero elements in the vector **x**. Invariance has been considered since correct decoding and object recognition are possible despite transformations induced to the sequence **b**^*i*^ (via the channel) prior to its entering the decoder. Works such as (Usher and Niebur, 1996) have advocated the IT exhibiting a larger overlap in its representations of similar objects than in its representation of dissimilar objects. The overlap of the similar objects is conveniently modeled in CTOR by such objects having decoded sequences that are relatively close in Hamming distance. Conversely, the decoding of dissimilar objects will result in sequences that have a larger discrepancy in Hamming distance. For instance, the representation of an object such as **b**^1^=brown bear is expected to be closer in Hamming distance to **b**^2^=baby elephant than to **b**^3^ = green hat. Inspection of a simple, synthetic example such as

(8) $$\begin{array}{r} \text{brown bear}:{\text{b}}^{1}=110010111001001\\ \text{baby elephant}:{\text{b}}^{2}=110010111001100\\ \text{green hat}:{\text{b}}^{3}=101100101100101 \end{array}$$

indicates that *d*(**b**^1^, **b**^2^) < *d*(**b**^1^, **b**^3^), and *d*(**b**^1^, **b**^2^) < *d*(**b**^2^, **b**^3^). In other words, the first two decoded sequences are closer to each other than either sequence is to the third. We note that the decoding accuracy is dependent not only on the decoder, but also the encoder and the channel properties. For instance, the robustness attribute can not be realized by the encoder and decoder alone because a channel with a very poor CQM would perturb the encoded representation to a degree that the decoder would be incapable of correctly untangling the object's identity.

**Figure 4:**
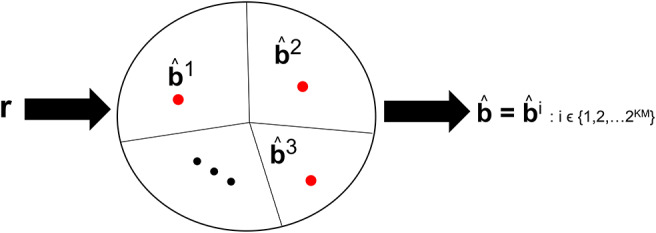
An example of the decision regions at the IT's decoder at a specific time instant. Suppose *KM* = 15 with 2^*KM*^ = 32, 768 possible representations that the decoder can declare as having been the viewed object. The circle denotes the possible space of received **r** sequences with **r** ∈ ℝ^*KN*^. The red dots denote the representation of such objects in a decision space. The closed region that surrounds a dot represents the region where the VVS would declare $\hat{\text{b}}={\text{b}}^{i}$ if **r** were to lie in that region. The sequences **b**^1^, **b**^2^, and **b**^3^ are three prototypical objects.

Visual processing works such as Reynolds and Chelazzi (2004) and Usher and Niebur (1996) have discussed the so-called competition among the neural representation of objects along the VVS. The competition occurs between a target object and distractors that are concomitantly present during the viewing. We posit that there is also competition among the objects stored in memory that are vying to be declared the viewed object. Such competition is incorporated in CTOR as the closeness among the decoded codewords. For example, in (8) there will be more competition among the representations **b**^1^ and **b**^2^ than among **b**^2^ and **b**^3^. Figure 4 depicts this notion with the closest messages competing within a decision space to be the representation associated with the viewed object. Models such as Usher and Niebur (1996) consider a suppression of the neural activity for a competing stimuli following a decision as to which object is present. With CTOR, the suppression of competitive stimuli occurs by the decoding operation discarding all prospective messages except for the selected $\hat{\text{b}}$. A comparative mechanism is inherent during the decoding operation since the codeword that is closest to the represented formulation is selected by the decoder as the decoded message. It was reported in Rust and DiCarlo (2010) that performance on visual discrimination tasks depend considerably on the number of neurons included in the analysis and the number of images included in the stimulus set. The decoding framework incorporates analogues for these two dimensions via the codeword length *N*, and the cardinality of the set of possible representations (i.e., |{**b**^*i*^}| = 2^*KM*^), respectively.

### 3.5. The Interleaver

**Figure 5:**
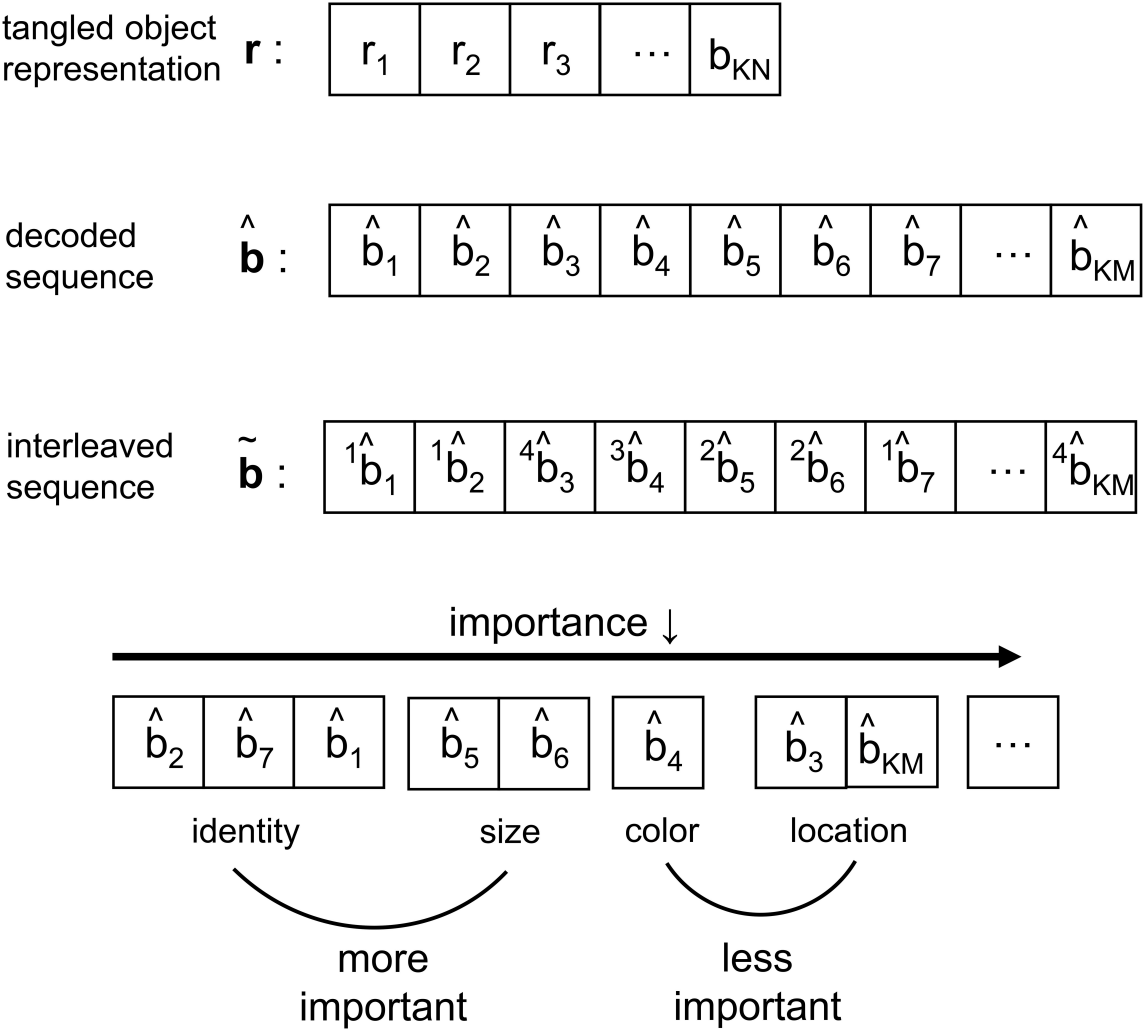
Depiction of the signal structure as it evolves along the VVS model presented in Figure 1. The *KN*-component vector **r** with *r*_*i*_ ∈ ℝ has been decoded into a message $\hat{\text{b}}$ with *KM* components, where ${\hat{b}}_{i}\in \left\{0,1\right\}$ and *N*≫*M*. At the output of the interleaver the *KM* components are partitioned into a message $\tilde{\text{b}}$ with *F* features—or feature dimensions as denoted in works such as Kanwisher and Wojciulik (2000)—of variable bit lengths. In the above example, *F* = 4 feature groupings are presented.

As the IT processes the representation from V4, the neural response is reformatted to be more selective for feature conjunctions (Rust and DiCarlo, 2010). In CTOR such processing is modeled via an interleaving operation. Interleavers are discussed in communication theoretic works such as Ramsey (1970), and have found application in computer science as well (Andrews et al., 1997). The biological motivation behind the interleaver lies in the necessity for the information output by the decoder to be parsed into a set representing the attributes and also the importance of the attributes for recognition. The interleaver shall arrange the decoder output into a sequence where the ordering has neurological significance for the efferent circuitry Since there is a need for considering the notion of feature grouping within the visual system (Olshausen, 2013), the interleaving operation in CTOR is a functional equivalent to the IT deciding the order of importance given to the features by consciousness and attention. In the example of the viewed object being a brown bear, the identity, size, color, and shape are ranked according to their importance. More important features such as identity appear before the less important features such as color (Figure 2). Figure 5 provides an example of the interleaving with the decoded message $\hat{\text{b}}$ being partitioned into smaller groups that correspond to the object's features. The ordering of the bits that comprise the interleaver output via the vector $\tilde{\text{b}}$ signify the order-of-importance of the features. This parsing and segmentation into components has been motivated by Biederman (1987). In Figure 5, the *KM* bits in the decoded message have been partitioned into *F* features with the variables *M*_1_, *M*_2_, …, *M*_*F*_ denoting the number of bits attributed to each feature. There is an obvious constraint that $\overset{F}{\underset{i=1}{\sum }}M_{i}=KM$. The *F* features that we allude to correspond to the stimulus dimensions introduced by the feature-integration theory of attention (Treisman and Gelade, 1980) that has been further elaborated upon in works such as Van Essen and Gallant (1994). Since the plasticity of the IT is responsible for refining the basic vocabulary of features (Serre et al., 2007; Rust and DiCarlo, 2010) it is expected that the interleaver is vastly distinct among different brains. It is also logical to posit that the interleaving operation is a highly dynamic process within a subject. With respect to neurophysiology, works such as Poggio and Kreiman (2013) and Meyers et al. (2008) have discussed the prefrontal cortex (PFC) guiding the IT (via a top-down signal) in the activation of subgroups of neurons to specific object features. It has been shown that PFC neurons also exhibit an increase in dendritic and spine complexity that is seen in the latter stages of visual cortical processing (Jacobs et al., 2001; Jacobs and Scheibel, 2002; Elston et al., 2011), and that the complexity is amenable to the progressive increase in sophistication of the computational operations. This was a motivation for the PFC-IT interaction considered in Figure 1 as the mechanism driving the interleaving operation. The interleaving operation constitutes a computation that is performed by populations of neurons acting collectively. Thus, the *M*-to-*M* component mapping of $\hat{\text{b}}\to \tilde{\text{b}}$ entails the coordinated firing among a population of neurons rather than the autonomous firing of neurons that may occur in populations at the lower visual layers. The output of the interleaver is comprised of *F* clusters with each cluster distinguishing a feature of the viewed object. In effect, the sequence $\tilde{\text{b}}$ is the information that the VVS has extracted (i.e., untangled) from the scene during object recognition via the decoding and interleaving operations.

### 3.6. Declaration of the Object Category

**Figure 6:**
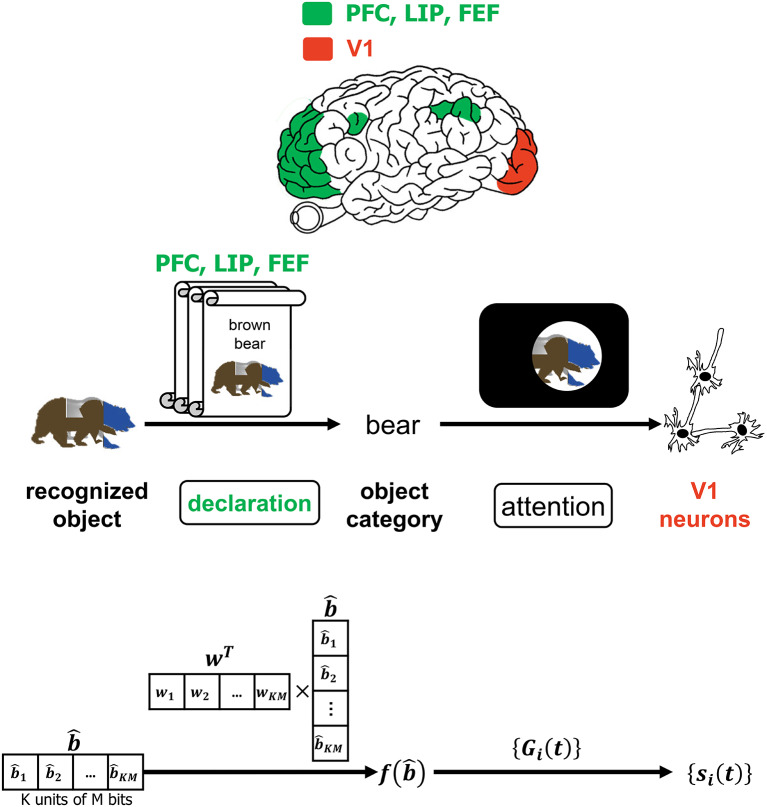
The interaction of attention and the declaration of object category. The top row depicts PFC, LIP, and FEF regions as they govern attention which is affected by the declared object category. The middle and bottom row provide an example of the CTOR operations and the dimensionality of the associated signals during the declaration of a viewed object's category. The scrolls denote different categories that viewed objects may be classified into prior to affecting attention. Attending to a categorized object is believed to modulate the firing rates of V1 neurons as discussed in Equation (1) and depicted via the projection (a) of Figure 1.

Despite the advancements in the study of primate vision, it has not been ascertained at what specific juncture in the VVS a viewed object can be said to have been recognized. The authors in (Neri and Heeger, 2002) advocate the presence of two stages in the VVS with the first performing object detection ~ 100ms prior to the second stage performing identification of the object's features. Figure 6 is a more detailed depiction of the operations associated with declaring the object category that was alluded to in Figure 1. In Figure 6, a classifier deciphers the object category by processing the decoded output. The CTOR model considers the progression of the decoder output into a classifier and an interleaver. Such parallel processing reflects the VVS's capability to classify the object category concomitant to discerning its features. A computationally simple model for object categorization is the inner product of the decoder output with a weight vector **w** via

(9) $$\begin{array}{l} f\left(\hat{\text{b}}\right)={\text{w}}^{T}\hat{\text{b}}. \end{array}$$

This is essentially the linear classifier readout advocated in (Rust and DiCarlo, 2010) although it is expected that the dimensionality $\text{dim}\left(\text{w}\right)=\text{dim}\left(\hat{\text{b}}\right)=KM$ for CTOR will be significantly larger than what has been previously considered. It is important to note that the output of *f*(·) is not sensitive to the order of the elements in the column vector $\hat{\text{b}}$ since **w** can be adjusted accordingly. Works such as Rust and DiCarlo (2010) and Pagan et al. (2013) have determined a realization of the vector **w** for every presented image in a set. While the selection of a classification technique for determining **w** is not the objective of this work, we remark on a crucial point. The assignment **w**^*T*^ = [1, 1, …, 1] would lead to the discernment of the object identity being solely a function of the Hamming weight of the decoded message. The above consideration for *f*(·) also instantiates CTOR as exhibiting the invariance attribute since the components in $\hat{\text{b}}$ can be re-arranged without a change in a declaration of the identified object's category. The distinction between an object's category and identity should be apparent. In the presented example, “brown bear” is the identity of the input object while “bear” is a declared category.

## 4. The Necessity of Attention, Compression, and Memory

Seminal works such as Biederman (1987) and Treisman and Gelade (1980) have motivated the importance of considering memory, attention, and object recognition within a unified model. The patent biological interplay between the aforementioned processes leads one to believe that an incomplete analysis would result by not considering such processes as interacting either via feedforward or feedback connections. The authors of Usher and Niebur (1996) have also advocated the concurrent consideration of attention and memory with the neural activity associated with the early visual stages. The model presented in the aforementioned work considers the necessity of a top-down feedback projection when a subject is searching for an expected target in a scene. This section will discuss how CTOR accounts for the interaction of attention and memory to provide a unified model for object recognition.

### 4.1. Attention as a Top-Down Modulatory Signal

The incorporation of attention as the modulator of the neural processes associated with object recognition is crucial. A review of the neural circuitry in the visual cortex that is actively modulated by attentional feedback has been presented in Reynolds and Chelazzi (2004). From analysis in monkeys it is natural to suggest that the attention module in Figures 1, 6 would contain the lateral intraparietal area (LIP) and frontal eye fields (FEF). CTOR posits attention as modulating components such as the encoder, channel, decoder, and compression via a top-down regulatory mechanism (Figure 6). Attention affects the encoder via the multiplicative factors {*G*_*i*_(*t*)} in (1) that drive the spiking rates of the retinal and V1 neurons. This reflects a role associated with the projection from the attentional circuitry to the encoder. A subject's attentional state will also influence the channel by affecting the conditional distribution *P*[**r**|**s**]. In the case of a continuous channel the effect may be seen on the SNR values {SNR_i_} which are a function of a subject's vigilance as well as the inherent neural noise along the VVS. It is sensible to assume that the SNR values increase with greater levels of attention. In the case of a discrete channel a similar modulation is expected with the conditional entropy values being affected by attention. The decoder is immanently influenced by a subject's attentional state through the vector **r** that the decoder must process during each epoch. This is seen by noticing that the transition metrics, path metrics, and survivor paths computed at the decoder en-route to declaring a message $\hat{\text{b}}$ are determined by the channel and the encoder. We have mentioned that with CTOR the number of bits attributed to each feature by the interleaver is a dynamic process modulated by attention. Works such as Cukura et al. (2013) and Huth et al. (2012) provide experiments that illustrate attention driving the degree of compression applied to what constitutes the *F* interleaved features in CTOR.

Attention also modulates the goals of object recognition. Consider the general scenario of a subject knowing that he/she must espy a scene before making a critical decision on an object in the scene. A nominal example of this is a driver checking a blind-spot immediately before changing lanes on a highway. The brain will have a snapshot view of the scene and, due to the heightened level of attention necessary for this task, perform object recognition much more quickly than during typical visual tasks. In such a pedestrian example the IT's decoder would recognize a car but the brain would allocate significantly more importance to the location and proximity of the car than its color or luminance. Brain imaging neuropsychological studies conducted in works such as Kanwisher and Wojciulik (2000) and Turk-Browne et al. (2013) have explored attentional modulation of visual encoding, memory formation, and the brain's capability to prioritize the sensory information that is most relevant for a task. It is necessary that a computational vision model also incorporate such notions. The CTOR model currently considers attentional selection by the increased firing of V1 neurons, while not accounting for the more sophisticated scenario of overlapping objects as described in works such as Baldauf and Desimone (2014). The incorporation of the biological functions associated with the capability of the VVS to separate attended and unattended objects is an avenue for the advancement of CTOR as its constituent portions are expanded upon. For instance, it can not be claimed that the entire VVS would consist of a single realization of Figure 1. Rather, it is more likely that there would be multitudes of such an architecture acting in parallel prior to a convergence. In Kersten et al. (2004), the parallel implementation of Bayesian models is mentioned and the authors advocate decomposing a scene or concurrently viewed objects into *m* features. **Figures 1, 2**, which have been a thrust of this work, will need to be cascaded into parallel streams to form a more comprehensive scheme that accounts for the case of overlapping objects competing for attention.

### 4.2. A Compressed Representation of Recognized Objects

**Figure 7:**
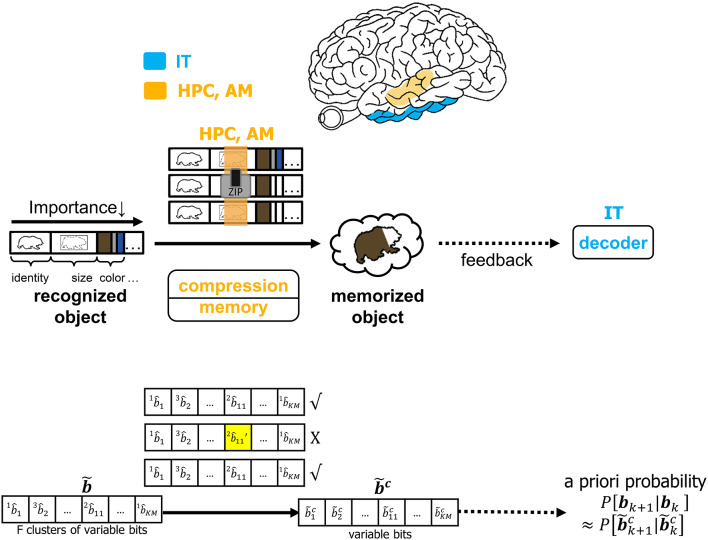
The operation of compression, memory and feedback in CTOR. The top row illustrates the brain regions (HPC, AM) involved in the memory and the compression operation while the middle row provides an illustrative example of the CTOR operations with brown bear being the viewed object. The bottom row depicts the dimensions of the neural signals during the aforementioned operations. The a priori probability *P*[**b**_*k*+1_|**b**_*k*_] that is fed back to IT is estimated as $P\left[{\tilde{\text{b}}}_{k+1}^{c}|{\tilde{\text{b}}}_{k}^{c}\right]$ from the compressed sequence ${\tilde{\text{b}}}^{c}$.

It is infeasible to conceive that the brain will commit every feature of each identified object to memory. The CTOR model allows for the incorporation of a compressive operation to proceed the interleaving process. The degree of compression will be a dynamic process modulated by attention and will shorten the representation of each object based on its most important features. This may be achieved by prioritizing the features that have been highly ranked by the interleaver while summarizing or even discarding the less-important features of a viewed object. Figure 7 depicts compression taking place in the hippocampus and amygdala where all objects' features such as identity, size, and color are subject to compression prior to being committed to memory. In CTOR, this process is achieved by combining multiple occurrences of similar objects into a single representation in memory as a sequence ${\tilde{\text{b}}}^{c}$. The memory circuitry is also driven by attention and will be presumed to have a fundamental role of providing the IT with the top-down a priori probabilities necessary for the IT to perform inference. The hippocampus's storage and rapid consolidation of object representations has been considered for decades with works such as O'Reilly and McClelland (1994) suggesting that the hippocampus is constructed to perform such a function. From a reverse engineering perspective, it is highly efficient that an object viewed at the highest frequency be allocated the smallest number of bits in memory. Different from the compression technique in CTOR, this alternative strategy would minimize storage and be akin to compression in the sense of Huffman coding or more recent proposals that suggest the hippocampus is performing even more sophisticated compression techniques (Petrantonakis and Poirazi, 2014).

### 4.3. The Consideration of Memory

For an object to be accurately recognized, a representation of the object must have been previously compressed and stored at an acceptable fidelity. There has been substantial evidence that memory-associated brain regions such as the hippocampus and amygdala are crucial for the neural processing underlying object recognition. Classical studies have referred to the area TE as containing “neural traces” associated with previously viewed stimuli (Mishkin et al., 1983). Such traces serve as stored representations against which subsequently viewed stimuli are compared. CTOR subsumes the comparisons into the decoding operation performed at the IT. The formation and storage of the traces are deemed as occurring at the hippocampus-amygdala circuitry that Figure 7 portrays communicating with the IT via feedforward and feedback connections. This is also illustrated in Figure 1 as the feedback connection from memory to decoder. The prevalence of the signaling from the memory circuitry to the IT and the neural circuits governing attention has also been justified in works such as Chelazzi et al. (1998) where the authors considered feedback provided by memory as a top-down signal for modulating the attention allocated to the object's attributes. The CTOR model considers two interactions between the memory and decoding circuitry that will propagate the transition probabilities {*P*[**r**_*k*_|**b**_*k*+1_, **b**_*k*_]} and the a-priori probabilities {*P*[**b**_*k*+1_|**b**_*k*_]} between the two entities. Firstly, the feedforward signal from the decoder that enters the hippocampus reflects memory formation following the recognition of an object and its associated features. Conversely, when a subject is processing a scene and attempting to recognize an object within the scene, the brain vests attention and draws upon stored memories to perform the recognition. It is expected that memory provides the a priori probabilities {*P*[**b**_*k*+1_|**b**_*k*_]} to the decoder during decoding (Figure 7). Works such as Olshausen (2013) have discussed the importance of feedback in the visual system as a potential means of communicating, via a top-down signal, the a priori probabilities that the brain uses when performing inference in stimulus space. The feedback connection considered by CTOR from memory is a means of enabling the decoder portion of the IT to operate in Bayesian fashion by providing the decoder with updated a priori probabilities. Secondly, object recognition can not occur without the IT having access to an itemized list of objects and attributes. We posit that such a dictionary exists and is continuously updated via the feedforward and feedback signaling discussed herein. The components of the dictionary are compressed versions of the previously viewed representations. The work of Mishkin has provided analytical motivation and experimental results on the notion of recognition memory. The interaction of the PFC in guiding working memory and visual search has also been considered in a model presented in Usher and Niebur (1996) that was further advocated in Poggio and Kreiman (2013). For a decoder at the IT to implement the Viterbi algorithm it must have knowledge of the encoder and the channel statistics. We can explain this as synaptic plasticity that occurs between neural populations of various brain regions that share connection. That is how an upstream population in IT could learn about some properties of the V4 and V1 neurons that constitutes the transition probabilities. In other words, the transition probabilities {*P*[**r**_*k*_|**b**_*k*+1_, **b**_*k*_]} must be conveyed to the decoder from the memory circuitry. The hippocampus and amygdala will continuously update their account of the transition probabilities by repeated interaction with the decoder in the IT. It is conceivable that during a developmental or training phase—that a subject may be agnostic to—the memory circuitry extensively communicates with the IT in order to update its estimates of the transition probabilities. Works such as Van Essen and Gallant (1994) and Miyashita (1993) have also cited IT neuron responses in primates as being markedly changed through repeated exposure to a limited set of stimuli. Accordingly, with CTOR the IT-hippocampus interaction will be an iterative process—if the decoded output is such that $\hat{\text{b}}\approx \text{b}$, then the VVS may maintain the transition probabilities as legitimate estimates for ensuing epochs until $\hat{\text{b}}$ deviates sufficiently from **b** (Kersten et al., 2004). In statistical communication theory the above procedure is referred to as the decoder learning the channel and is implemented via means such as the Baum–Welch algorithm (Hastie et al., 2009).

## 5. The Operation of CTOR

It is insightful to consider an example of CTOR operation that commences with the tangling of the stimulus representation and concludes with a decoding, interleaving, and commitment to memory of the untangled object identity. We consider an example where at each time instant the early visual stages will tangle *M* = 1 bits of the object identity into a *N* = 3 bit sequence. We also consider *K* = 4 and thus the identity of the viewed objects will lie in a space with a cardinality of 16. As part of this toy example, suppose that the viewed object has the representation **b** = 1100 at the IT. Of course this constitutes a highly synthetic stimulus signal with *M*, *N*, and *K* values small enough for the analysis to be tractable while still elucidating the computations advocated by CTOR. We caution that although computational intractability is avoided in this example, it is by no means reflective of the VVS avoiding such intractabilities—obviously the VVS's prospective implementation of the encoding and decoding would encompass significantly larger *K* and *N* values. The interleaving, categorization, and compression operations will also be instantiated in the toy example of this section.

### 5.1. Object Tangling and Manifestation of the Channel

From a biologically-inspired perspective, an encoder of rate 1/3 signifies that every bit from the representation that the viewed object should evoke at the IT has been tangled by the retina, LGN, and V1 into three bits. We shall consider the encoder in Figure 3A since it has already been discussed in section 3.2. Communication theorists would describe this encoder via a so-called algebraic generator sequence *G*(*D*) = [1 + *D*, 1 + *D*^2^, 1 + *D* + *D*^2^] and recognize that the encoder has a maximal memory of *L* = 2 that allows the encoder to take 2^*L*^ = 4 possible states at each time instant. The encoder in Figure 3A has been extensively discussed in Lin and Costello (1983) and will be dubbed “simple-encoder” for the remainder of the paper. For clarity, the four states shall be referenced via {*S*_0_, *S*_1_, *S*_2_, *S*_3_} as shown in the state diagram in Figure 3B. The number of possible transitions in the encoder state diagram is 2^*N*^ = 8. For instance, *S*_0_ → *S*_2_:111, 1 and *S*_0_ → *S*_0_:000, 0 denote two of the transitions. The *N* = 3 bits written above each transition is the encoder output that is generated due to the combination of that transition and the *M* = 1 bit input to the encoder (e.g., “1” and “0” for the *S*_0_ → *S*_2_ and *S*_0_ → *S*_0_ transitions, respectively). The encoding operation has structure that is modeled via a state-machine - this reflects that the tangled signals converging at the IT via afferent projections are not completely random patterns. For instance, regardless of the nature of the viewed object, it is obvious that the encoder in Figure 3 would prohibit an encoded sequence of 111 to be followed by an encoded sequence of 001. It can be verified that the considered sequence **b** = 1100 would be encoded into **s** = 111 010 110 011 according to the state diagram of simple-encoder.

During the encoding or tangling operation the neural representation that a viewed object should evoke at the IT is perturbed by a channel that encompasses the visual impairments inherent to the scene as well as neural noise inherent to the VVS. We model this via each element in **s** being stochastically transformed into one of four values denoted by A, B, C, and D. The four values reflect different intervals for the neural activity produced by the circuitry that projects to the IT. Consider the discrete memoryless channel quantified by the following conditional probabilities:

(10) $$\begin{array}{l} P\left[r_{i}=A|s_{i}=0\right]=0.4\\ P\left[r_{i}=B|s_{i}=0\right]=0.3\\ P\left[r_{i}=C|s_{i}=0\right]=0.2\\ P\left[r_{i}=D|s_{i}=0\right]=0.1\\ P\left[r_{i}=A|s_{i}=1\right]=0.1\\ P\left[r_{i}=B|s_{i}=1\right]=0.2\\ P\left[r_{i}=C|s_{i}=1\right]=0.3\\ P\left[r_{i}=D|s_{i}=1\right]=0.4. \end{array}$$

The above is one of the channels considered in Lin and Costello (1983) and it is used in this illustrative example for its relative simplicity—it is easy to verify that *H*(*r*_*i*_|*s*_*i*_ = 0) = *H*(*r*_*i*_|*s*_*i*_ = 1) = 1.846 bits. The channel output is presumed to be the sequence

(11) $$\begin{array}{l} \text{r}=\left(DCA,DDB,DDA,DDD\right). \end{array}$$

The decoder considered in the following section will process the sequence **r** via the Viterbi algorithm in order to attain a MAP estimate of the viewed object's representation. The accuracy of the recovery process will quantify the fidelity at which the object identity **b** has been untangled at the IT.

### 5.2. Decoding Dynamics

**Figure 8:**
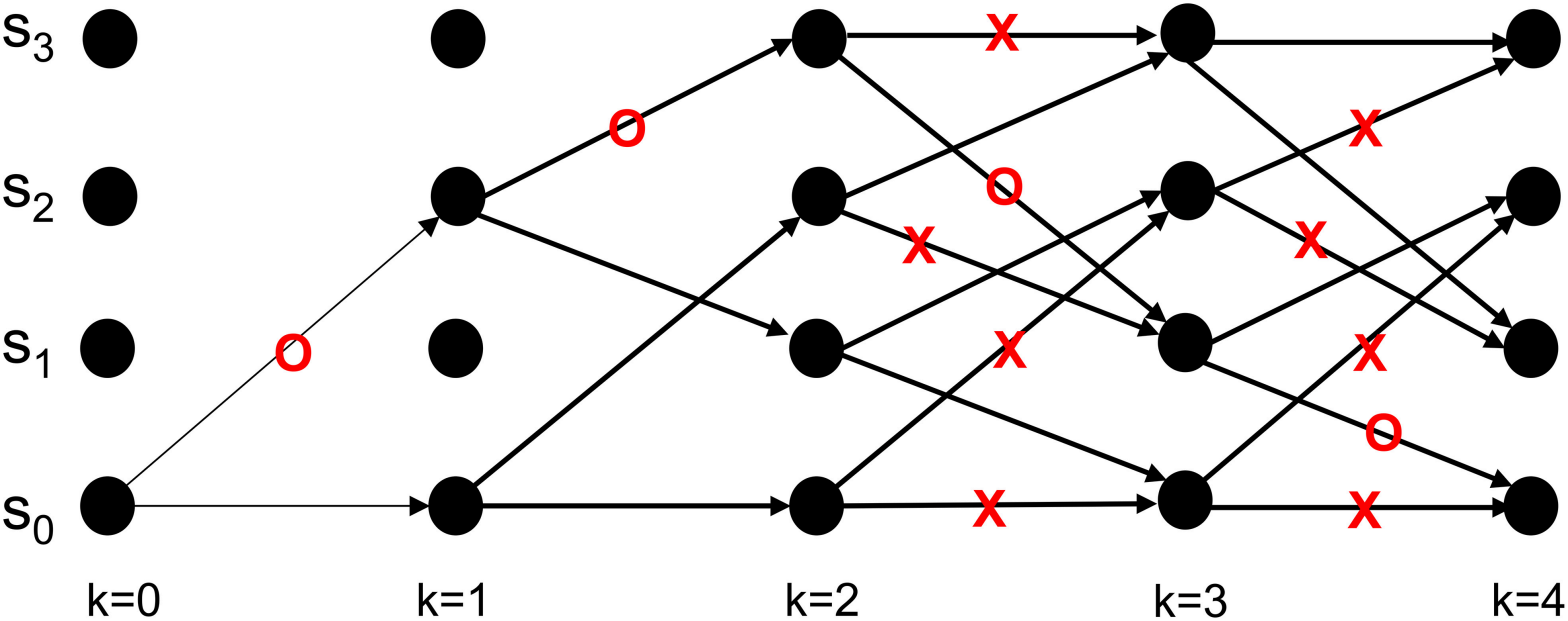
An example from Lin and Costello (1983) to illustrate the dynamics of Viterbi decoding. The path marked with “O” denotes the final survivor path selected based on the smallest path length which corresponds to the MAP estimate of the sequence that the encoder desired to convey to the decoder. The transitions marked with “X” denote non-survivor transitions, while the unlabeled transitions denote survivors that were not part of the final survivor path.

Neural activity at the IT is believed to correspond to the untangled identity of the object that has been communicated to the IT (in tangled form) by the lower layers of the VVS. Cortical computation presentations such as Rao and Ballard (1999), Olshausen (2013), Lee and Mumford (2003), and Kersten et al. (2004) have advocated a hierarchical Bayesian model with top-down and bottom-up information flow. Such dynamics are at the heart of proposed decoding operation for the IT. The decoder uses bottom-up information from the encoder in conjunction with top-down information from memory to recover the object identity. The top-down information quantified by the a priori probabilities {*P*[**b**_*k*+1_|**b**_*k*_]} will be assumed as uniform (i.e., equally-probable) among the different competing stimuli representations, and thus will not affect the transition lengths in (6). The decoding procedure applied to the sequence in (11) is shown via the trellis diagram of Figure 8 with the decoder's initial condition given by **b**_0_ = *S*_0_. At time *k* = 1 the decoder computes the transition lengths via (6) as

(12) $$\begin{array}{l} \lambda \left({\text{b}}_{1}=S_{0},{\text{b}}_{0}=S_{0}\right)=-\ln\left(P\left[DCA|{\text{b}}_{1}=S_{0},{\text{b}}_{0}=S_{0}\right]\right)\\ \;=-\ln\left(P\left[r_{1}=D|\text{input}=0\right]\right)\\ \;-\ln\left(P\left[r_{1}=C|\text{input}=0\right]\right)\\ \;-\ln\left(P\left[r_{1}=A|\text{input}=0\right]\right)=4.82\\ \lambda \left({\text{b}}_{1}=S_{2},{\text{b}}_{0}=S_{0}\right)=-\ln\left(P\left[DCA|{\text{b}}_{1}=S_{2},{\text{b}}_{0}=S_{0}\right]\right)\\ \;=-\ln\left(P\left[r_{1}=D|\text{input}=1\right]\right)\\ \;-\ln\left(P\left[r_{1}=C|\text{input}=1\right]\right)\\ \;-\ln\left(P\left[r_{1}=A|\text{input}=1\right]\right)=4.42.\; \end{array}$$

The two possible transitions above are considered by the Viterbi algorithm because of the encoder's state diagram in Figure 3B. It should be noted that at *k* = 1 there are two rather than 2^*L*^ = 4 survivors because the decoding has just commenced. Subsequently, the decoder computes

(13) $$\begin{array}{l} \Gamma \left({\text{b}}_{1}=S_{0},{\text{b}}_{0}=S_{0}\right)=\Gamma \left({\text{b}}_{0}=S_{0}\right)+\lambda \left({\text{b}}_{1}=S_{0},{\text{b}}_{0}=S_{0}\right)=0+4.82\\ \Gamma \left({\text{b}}_{1}=S_{2},{\text{b}}_{0}=S_{0}\right)=\Gamma \left({\text{b}}_{0}=S_{0}\right)+\lambda \left({\text{b}}_{1}=S_{2},{\text{b}}_{0}=S_{0}\right)=0+4.42. \end{array}$$

This process is repeated for time *k* = 1, 2, ...*K* (see the steps in Appendix B). At *k* = *K* the Viterbi algorithm proceeds backwards in the trellis of Figure 8 to arrive at (**b**_1_ = *S*_2_, **b**_0_ = *S*_0_), (**b**_2_ = *S*_3_, **b**_1_ = *S*_2_), (**b**_3_ = *S*_1_, **b**_2_ = *S*_3_), (**b**_4_ = *S*_0_, **b**_3_ = *S*_1_) as the final survivor path because it has the smallest metric among all of the candidates. The decoded sequence corresponding to this survivor path is

(14) $$\begin{array}{l} \hat{\text{s}}=111\;010\;110\;011 \end{array}$$

which corresponds to the decoder's estimate of the encoded message being

(15) $$\begin{array}{l} \hat{\text{b}}=1100. \end{array}$$

By decoding, the stream **r** in the above example into the sequence $\hat{\text{b}}$, the IT has untangled the object's representation that was propagated along the VVS. In the above example $\hat{\text{b}}=\text{b}$ which indicates perfect object recognition at the IT.

The assignment of uniform priors to the metric in (6) has the biological ramification of the IT having no prior memory, or synonymously, an unbiased account of what object to expect. If the VVS were to have identified an object in the prior *K* = 4 discrete time instances, then it would be sensible for the IT to have non-uniform priors with the first term in (6) biasing the transition metrics toward a particular representation. In organizing the CTOR model to emulate VVS operation, an updating rule should be presented to adjust the priors based on the object that was decoded in previous epochs, or is expected during the current viewing interval. As discussed in section 4.3, the a priori probabilities will be communicated from memory to the decoder (via the feedback signal in Figure 1) to be used in the ensuing decoding. Formulation of an updating rule for the priors that are stored in memory is an important future avenue because it would further substantiate the model's biological feasibility.

Properties such as poor visibility and a subject's inattention are factors that can adversely affect the decoding process by bringing about a channel with a low CQM. This will affect the decoding process in a conspicuous manner regardless of the decoder's proficiency. For instance, consider a case where (10) is replaced with the following channel

(16) $$\begin{array}{l} P\left[r_{i}=j|s_{i}=0\right]=0.25\\ P\left[r_{i}=j|s_{i}=1\right]=0.25\text{ for }j=A,B,C,D \end{array}$$

that has a conditional entropy of *H*(*r*_*i*_|*s*_*i*_ = 0) = *H*(*r*_*i*_|*s*_*i*_ = 1) = 2 bits. Assuming uniform priors, it can be confirmed from (6) that the above channel would yield transition metrics of equal value at each time instant in the decoding process. The consequence of this is that the IT will have no choice but to arbitrarily select one of the possible 1/2^*KM*^ sequences. The preceding is an example of how recognition can be obscured by a catastrophically bad channel. The properties associated with the stimulus, environment, and neural circuitry that may bring about such a channel are not immediately obvious, but this is a question that warrants scrutiny. Following the decoding operation it is possible that the IT is indecisive as to the stimulus identity. In such a case the VVS may declare an erasure (Forney, 1968) as a means of requiring additional time to decide upon the identity or attributes of the viewed object. From a psychophysics perspective it is expected that the erasure is reflected by a higher reaction time and degraded processing speed for recognizing the object. The dynamics and threshold associated with the declaration of an erasure by the IT after decoding is an avenue for future consideration. It is interesting to note that an erasure may not be a complete waste of time and resources by the VVS since information may be gained and used about the viewed object at subsequent time epochs. This is expected of an adaptive system that has been optimized through continuous training and evolution.

### 5.3. Declaration of Object Category

In this example, we assume **w**^*T*^ = [1, 1, 1, 1] which would result in $f\left(\hat{\text{b}}\right)={\text{w}}^{T}\hat{\text{b}}=2$ via (9). This operation is perhaps too elementary in this toy example because we except more than four object categories to exist during the viewing of a stimulus. It is more insightful to examine the scenario given by (8). The assignment of **w** as a 15-dimensional vector of 1's yields $f\left(\hat{\text{b}}\right)=8$ for the three decoded sequences of (8), and hence the three stimuli would be categorized into the same category. There are three important points that follow with respect to this dubious outcome. First, although the three objects would be classified under the same category, their differing features can be still discerned by the IT assuming a sufficient degree of redundancy at the encoder, a channel that is not too dispersive, and adequate processing at the decoder. Second, the choice of **w** has not been determined via an SVM or even a correlation-based classifier as considered in works such as Rust and DiCarlo (2010) and Meyers et al. (2008), respectively. Both techniques would provide a **w** that has been acquired via a training process on already-viewed stimuli. For instance, it is easy to confirm that the (non-unique) choice **w**^*T*^ = [1, 0, 1, 1, 0, 1, 0, 1, 0, 0, 1, 0, 0, 1, 1] would yield perfect classification of category for this example. Third, a more biologically realistic scenario would have a significantly larger *N* value. This would lead to greater granularity among the object categories and significantly better classification capability.

### 5.4. Interleaver Operation, Message Compression, and Memory

The interleaving operation is considered as a means for identifying the important features in the decoded sequence. In this example we consider two features via *F* = 2 with *M*_1_ = 2 and *M*_2_ = 2. The static interleaving operation given by I=[[1,3],[2,4]] will be assumed where [1, 3] signifies the bit indices corresponding to the more important feature *M*_1_, and [2, 4] refers to the bit indices of a less important feature *M*_2_. Thus, the interleaving will lead to the following grouping

b^=1100→b˜=[b^11,b^22,b^13,b^24]=11120102

where the left superscript of each bit indicates its importance level as dictated by the interleaver. The operation I is equivalent to a mapping that ranks the importance of the bits in the decoded stream via the features that they correspond to. In the present example, the more important feature, *M*_1_, represents the object identity while *M*_2_ will correspond to the object's size. As shown in Figure 1, the interleaving operation is dynamically guided by attention in the prioritization of sensory information that is necessary for a task. To consider an instance of compression, suppose that the same object is presented to a subject during the next three viewing intervals with the corresponding representation given by $\hat{\text{b}}=1100\;1100\;1110\;1101$. The third representation has a different object identity than the other three while the fourth representation differs from the first two due to the object's size varying as a result of a change in viewing distance. The 16-bit decoded version of this sequence $\hat{\text{b}}$ would be interleaved to $\tilde{\text{b}}$. With the hippocampus performing a compressive operation, the most important features of the representation $\tilde{\text{b}}{=}^{1}1^{2}1^{1}0^{2}0{\;}^{1}1^{2}1^{1}0^{2}0{\;}^{1}1^{2}1^{1}1^{2}0{\;}^{1}1^{2}1^{1}0^{2}1$ are committed to memory with less compression than the less-important features. More specifically, the features with a left superscript of “1” are 10 and 11 while the less important features have been labeled via a superscript of “2,” i.e., 10 and 11. A compression mechanism may entail the less important features being compressed among viewed objects that share the same important features. In the present example, the first and second representations 1100, and the fourth representation 1101 share the same value of important feature 10. These three objects can be compressed in memory as 1100 since it appears more frequently than 1101. The other value for the important feature (i.e., 11) occurs only in the third viewing of the object via 1110 which is stored in memory as well. Thus, for the considered compression, the 16-bit input $\hat{\text{b}}=1100\;1100\;1110\;1101$ is compressed into the 8-bit representation ${\tilde{\text{b}}}^{c}=1100\;1110$.

Having attained a compressed representation of the viewed object, it is possible for the a priori probabilities {*P*[**b**_*k*+1_|**b**_*k*_]} to be computed at the hippocampus and amygdala. The neural circuitry can estimate *P*(**b**_*k*+1_ = *S*_1_|**b**_*k*_ = *S*_3_) from the sequence ${\tilde{\text{b}}}^{c}=1100\;1110$ by counting the occurrences of *S*_1_ = 10 after *S*_3_ = 11 and normalizing that value by the occurrences of *S*_3_ = 11. In the present example, *S*_3_ = 11 occurs three times with two occurrences followed by *S*_1_ = 10, therefore *P*(**b**_*k*+1_ = *S*_1_|**b**_*k*_ = *S*_3_) = 2/3. A more descriptive analysis of how the VVS may perform such a calculation for the remaining a priori probabilities is provided in Appendix C.

## 6. The Performance of CTOR, and Use of Prior Knowledge in Object Recognition

The previous section provided an instantiation of CTOR operation. It is also necessary to have an idea of the performance that is possible with this model. Accordingly, a more realistic scenario must be considered than the toy example of the previous section; clearly a larger stream is present as the input to the primate VVS. Since CTOR is not based on a neural network or a SVM, the metrics used to assess the performance of models based on the aforementioned methods are by-in-large not applicable here. To assess the performance of CTOR, several metrics must be discussed within the object recognition paradigm.

- *Bit Correct Rate (BCR)*: According to (7) the Hamming distance $d\left(\hat{\text{b}},{\text{b}}^{i}\right)$ was derived between the decoded sequence at the IT and the representation that the object should induce at the IT. The expression
  (17) $$\begin{array}{l} \text{BCR}=1-\frac{1}{T}\overset{T}{\underset{t=1}{\sum }}{\left[\frac{d\left(\hat{\text{b}},{\text{b}}^{i}\right)}{KM}\right]}_{t} \end{array}$$
  provides a measure of the deviation between the expected and decoded representations over *T* viewed sequences. In the above expression [*X*]_*t*_ denotes the value of the argument *X* at the *t*-th iteration. It is not difficult to observe that at chance BCR = 1/2.
- *Symbol Correct Rate (SCR)*: A more stringent measure of correct object recognition is given by
  (18) $$\begin{array}{l} \text{SCR}=\frac{1}{T}\overset{T}{\underset{t=1}{\sum }}{\left[𝟙\left(d\left(\hat{\text{b}},{\text{b}}^{i}\right)=0\right)\right]}_{t}. \end{array}$$
  In the above expression 𝟙(·) denotes the indicator function and the condition inside the indicator function is only satisfied for perfect recovery of the object identity. It can be verified that at chance SCR = 1/2^*KM*^.
- *Category Correct Rate (CCR)*: A distinction between object recognition and categorization has been made in the presentation of CTOR. Accordingly, we consider a measure for the correct identification of object category via
  (19) $$\begin{array}{l} \text{CCR}=\frac{1}{T}\overset{T}{\underset{t=1}{\sum }}{\left[𝟙\left({\text{w}}^{T}\hat{\text{b}}={\text{w}}^{T}{\text{b}}^{i}\right)\right]}_{t}. \end{array}$$
  It should be apparent that the classification vector **w** used in (19) is derived based on a classifier cost function rather than the CCR metric, otherwise the trivial solution **w** = **0** would result. At chance this metric will equal the reciprocal of the number of categories considered i.e., CCR = 1/*KM*.
- *Approximate Category Correct Rate (ACCR)*: A less stringent measure of categorization accuracy follows from considering the metric
  (20) $$\begin{array}{l} \text{ACCR}=\frac{1}{T}\overset{T}{\underset{t=1}{\sum }}{\left[𝟙\left(|{\text{w}}^{T}\hat{\text{b}}-{\text{w}}^{T}{\text{b}}^{i}|\leq c_{1}\right)\right]}_{t}. \end{array}$$
  The constant *c*_1_ > 0 is the maximum tolerable difference between the expected and recovered representation for the category to be determined at an acceptable fidelity. By its definition it can be noted that AACR ≥ CCR.

The performance of the CTOR model for the VVS will be analyzed for all of the aforementioned metrics. We are, in effect, attempting to justify the utility of the BCR, SCR, CCR, and ACCR within the object recognition paradigm. It is interesting that the presented dialogue has provided a means to quantitatively decipher between categorization performance and object recognition performance. To the best of our knowledge prior works have not made such a quantitative distinction and this may be viewed as a void in object recognition models.

**Figure 9:**
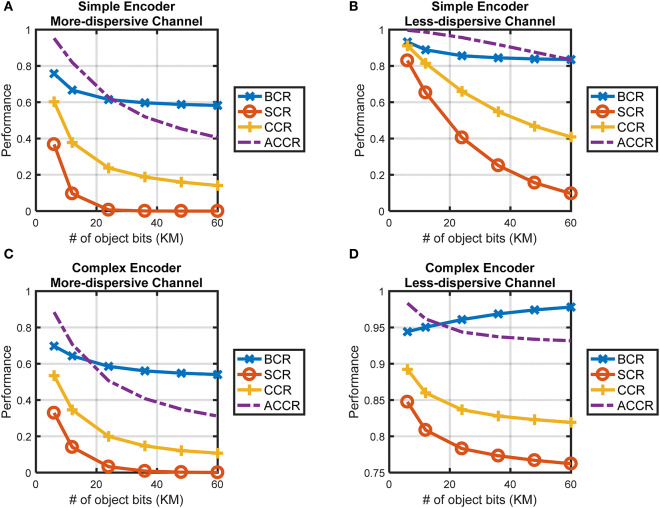
The performance of CTOR with the encoding, channel, and decoding structures discussed in this work. **(A)** The performance attained with simple encoder and the more-dispersive channel in (10), and **(B)** with the less-dispersive channel in (21). The decoding for the simple encoder was implemented with custom code in MATLAB. **(C)** The performance attained for complex encoder with the more-dispersive channel, and **(D)** with the less-dispersive channel. The decoding for the complex decoder was implemented using MATLAB's vitdec(·) function with 4 levels specified in the Viterbi soft-decision decoding algorithm.

The performance of CTOR with the simple-encoder in Figure 3A and its corresponding decoder implementing the Viterbi algorithm will be analyzed via simulation. The object representation **b** will be comprised of *K*= 6, 12, 24, 36, 48, or 60 bits meaning that the encoder will entangle such representations into a sequence **s** consisting of 3*K* bits. Without loss of generality, we specify the viewed stimulus as having a representation at the IT given by an alternating sequence of 1 and 0, e.g., for *K* = 6, **b** = 101010. For each object *T* = 10^6^ iterations will be considered in a Monte-Carlo (MC) simulation. Each iteration entails the components of the encoded sequence being probabilistically perturbed by the channel (10). The dispersive nature of (10) will be shown by examining object recognition in a less-dispersive discrete memoryless channel where the transition probabilities are given by

(21) $$\begin{array}{l} P\left[r_{i}=A|s_{i}=0\right]=0.65\\ P\left[r_{i}=B|s_{i}=0\right]=0.2\\ P\left[r_{i}=C|s_{i}=0\right]=0.1\\ P\left[r_{i}=D|s_{i}=0\right]=0.05\\ P\left[r_{i}=A|s_{i}=1\right]=0.05\\ P\left[r_{i}=B|s_{i}=1\right]=0.1\\ P\left[r_{i}=C|s_{i}=1\right]=0.2\\ P\left[r_{i}=D|s_{i}=1\right]=0.65 \end{array}$$

and lead to *H*(*r*_*i*_|*s*_*i*_ = 0) = *H*(*r*_*i*_|*s*_*i*_ = 1) = 1.416 bits. Figures 9A,B contain simulation results of the object recognition metrics for CTOR with simple-encoder and the channels given by (10) and (21). A value of *c*_1_ = 1 was used when computing the ACCR metric, in other words, a disagreement in Hamming distance of one between $\hat{\text{b}}$ and **b**^*i*^ was deemed tolerable in the recovery of the object category. The representation of more complicated stimuli would require a larger number of bits and may result in a degradation in the VVS's capability to accurately perform object recognition and classification. It is also expected that the more complex stimuli will require increased amounts of neural processing leading to longer message lengths (i.e., larger *K* values). The more convoluted an object, the worse a subject's performance in recognizing, categorizing, and parsing the attributes of the object. The CTOR model is capable of reflecting this aspect that seems fundamental to the working of the VVS. Indeed, the BCR, SCR, CCR, and ACCR metrics in Figures 9A,B show a degradation with increasing *K* values. It is interesting that the SCR shows the most precipitous degradation with increasing object complexity. This is attributed to the correct decoding of the entire object representation being more difficult, and hence more sensitive to the viewed object complexity, than a partial or a category-only recovery. A comparison of the four metrics shown in Figures 9A,B confirm an improvement in recognition and categorization performance when the channel is represented by (21) instead of (10). Thus, the impact of a degradation in CQM on object recognition is patent since the recognition and classification accuracies are lower for the more dispersive channel. The consideration of the retina, LGN, and V1 stage via a more sophisticated encoder shall be referred to as “complex-encoder.” The rate of the encoder is maintained at 1/3, but a maximal memory order of *L* = 8 is considered via the following generator sequence

$$\begin{array}{l} G(D)=[1+D^{2}+D^{3}+D^{5}+D^{6}+D^{7}+D^{8},1+D+D^{3}+D^{4}\\ \;+D^{7}+D^{8},1+D+D^{2}+D^{5}+D^{8}]. \end{array}$$

The above encoder has been studied in Lin and Costello (1983); its shift-register structure and state diagram are not shown because of their involved nature in comparison to simple-encoder. For instance, the decoding would consist of a trellis with 2^8^ = 256 states at time *k* and two prospective transitions out of each state. Via a higher constraint length (*L*), there are a larger number of paths to compare at each stage of the trellis and this leads to an increase in resolution when making a decision on every encoded bit. Thus, a decoder that would accommodate complex-encoder will generally be more accurate in recovering representations than the decoder accommodating simple-encoder. The performance of CTOR with complex-encoder shall now be assessed. Due to the increased complexity and run time, rather than using the complete Viterbi algorithm that was used for simple-encoder, MATLAB's vitdec(·) function with soft-decision decoding and 4 levels (i.e., nsdec = 2) were used in the simulations with complex-encoder. It shall still be assumed that the viewed object has a representation at the IT given by an alternating sequence of 1 and 0. The evaluation of the BCR, SCR, CCR, and ACCR in Figures 9C,D show that similar conclusions can be drawn for CTOR with complex-encoder as with simple-encoder. With the exception of the BCR for the channel of (21), the metrics in Figure 9 exhibit a degradation with increasing *K* values.

There are findings to discuss in light of the simulation results shown in Figure 9. The BCR appears to be the most robust of the metrics with respect to increasing degrees of stimulus complexity. By definition the BCR is restricted to the interval [0.5, 1], and the observed limited range in comparison to the other metrics in the simulations indicates that the BCR may not be as insightful of a metric. A comparison of the performance of simple- vs. complex-encoder shows that the latter exhibits a clear improvement across all of the metrics for the less-dispersive channel. Interestingly, the affect of the channel is more pronounced on the metrics for complex-encoder than for simple-encoder. In the case of the highly dispersive channel, however, the two systems yield similar performance. This is attributed to the increased processing not being able to overcome the detriments brought forth by the high dispersion. For an engineered system, so long as the channel is not overly dispersive, a higher *L* is desirable because it yields more reliable communication (i.e., higher BCR and SCR), the tradeoff is that an increase in constraint length leads to an increase in complexity and processing. Of course the VVS is not subject to the same tradeoffs that exist in engineered systems, thus it may be presumed that a CTOR implementation of the VVS will entail a large *L* value and accommodate the decoder (i.e., IT circuitry) being able to process an immense number of states in the prospective trellis.

**Figure 10:**
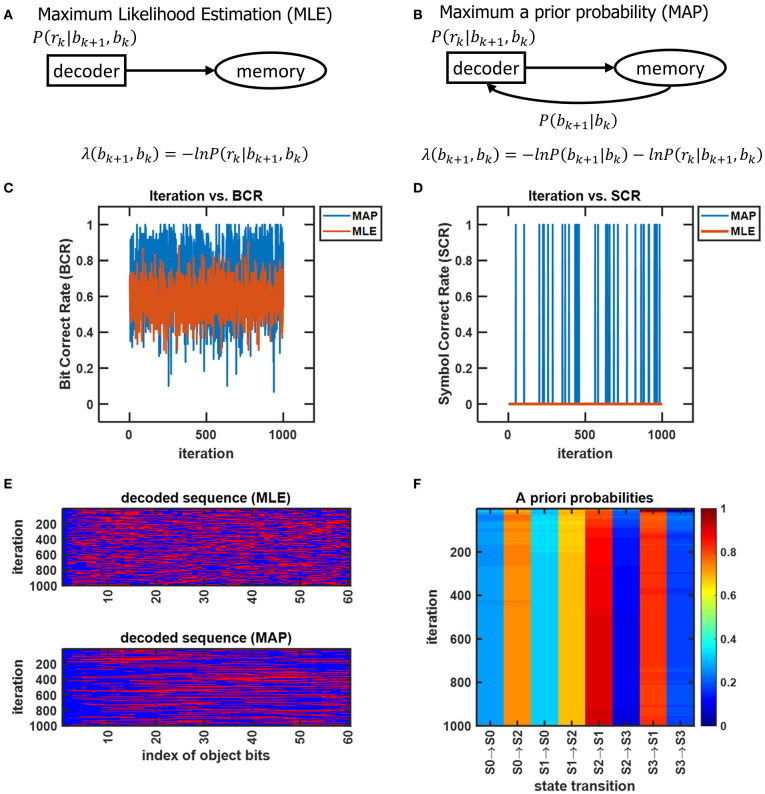
A comparison of MLE and MAP with a simple encoder, the dispersive channel of (10) and *K* = 60 bit object identity. **(A,B)** Algorithmic depictions of the MLE and MAP decoding schemes. The transition length λ(·, ·) is computed for both schemes, and for MAP decoding, includes the a priori probability *P*[**b**_*k*+1_|**b**_*k*_] in addition to the transition probability *P*[**r**_*k*_|**b**_*k*+1_, **b**_*k*_]. **(C)** The progression of the BCR at each iteration of CTOR when considering MLE and MAP for the same viewed object. **(D)** The progression of the SCR at each iteration of CTOR when considering MLE and MAP for the same viewed object. It should be noted that the SCR can only take on values of {0, 1} at any iteration. **(E)** A kymograph of the decoded sequence over different viewing intervals (i.e., iterations). Red represents a bit correctly identified by the decoder and blue indicates that the bit was erroneously identified. **(F)** A kymograph of the a priori probabilities calculated by the attention and memory circuitry over the duration of each viewing interval.

The operation of the CTOR model has been studied with the view of uniform a priori probabilities for the state transitions that are used at the IT for untangling the representation of the viewed object. While this has been done for ease in presentation, such an assumption reduces maximum a posteriori probability (MAP) decoding to maximum likelihood estimation (MLE). We shall now consider the scenario of non-uniform a priori probabilities for the state transitions. The simple encoder, more dispersive channel in (10), and an input sequence of *K* = 60 bits shall be assumed in considering the Viterbi algorithm operation with MLE (Figure 10A) and MAP (Figure 10B) decoding. The simulations were performed in the same manner as in Figure 9 except *T* = 10^3^ iterations were considered for each object. With MAP decoding, the a priori probabilities are estimated from the previous iteration using the technique described in section 5.4. The BCR attained with MLE fluctuates between 0.4 and 0.8 and has a mean of 0.581, while the mean BCR attained with MAP is 0.695 with the BCR equating to 1 at several iterations (Figure 10C). The SCR results show a similar trend as the MLE does not correctly recover the entire object at any iteration (i.e., mean SCR = 0) whereas MAP is able to do so (mean SCR = 0.032) (Figure 10D). A kymograph of the decoded bits across each iteration illustrates that the correctly recovered bits are more clustered for MAP decoding than with MLE (Figure 10E). This is because knowledge of the a priori probabilities guides the fidelity with which consecutive bits are decoded. It is observed that the a priori probabilities computed at the decoder during MAP decoding are rather constant across the 10^3^ iterations (Figure 10F). This is expected because the same input sequence was used for each iteration. Furthermore, we note that the state transitions *S*_2_ → *S*_1_ and *S*_3_ → *S*_1_ are assigned the highest a priori probabilities. This is also expected since it can be verified that the transitions 11 → 10 and 01 → 10 will be the most frequent transitions for the considered input stream. In summary, the CTOR formulation with MAP decoding surpasses the performance noted with MLE (Figures 10C,D), thus confirming the value of the feedforward-feedback interaction between the IT and hippocampus during object recognition.

## Conclusion

Three communities are concurrently involved in the comprehension of visual object recognition: neuroscientists, computer vision scientists, and visual psychophysicists. The presented CTOR model has drawn upon elements advocated from the three realms. Previously considered for lower visual areas, dynamic inference via an on-line algorithm for MAP sequence estimation has been proposed for the higher visual areas implicated during object recognition. Although the primary motivation for CTOR is to provide an account for the proficiency of the IT, the formulation is also a starting point for a more comprehensive scrutiny of the computations performed by the VVS during real-time object recognition. The performance of the model was evaluated by presenting several metrics to assess categorization accuracy and object identity recognition. The simulation results provide insight into the dynamics and capabilities of CTOR. The role of attention and memory have been incorporated via top-down signaling that guides the inference, and is also affected by the cognition. Empirical corroboration of CTOR would entail presentation of data to support or verify the algorithmic notions discussed in this work. In order to test or affirm aspects of CTOR in the framework of current knowledge, it is crucial to consider primate neuroscience studies that have already amassed high-dimensional recordings from multiple brain regions and pursued computational questions. The study in Shinomoto et al. (2009) considers neural spike data from 15 cortical areas in awake, behaving monkeys that were collected at different labs. The authors used this data to make statements about the functional category of the cortical area. A similar methodology can be used to assess aspects of CTOR. For instance, an experiment could entail showing the same objects to subjects, recording V1, V4, and IT neural responses, and amassing the collected data among different labs into one dataset to evaluate the encoding and decoding operations. Initially, the IT neural population responses would be compared to the V1 responses in order to determine the encoder. In effect, a code rate, constraint length, and encoder structure would be assumed, evaluated, and altered in iterative fashion until a candidate has been deemed as fitting the data appropriately. Such iterative searches are routinely performed by coding theorists—e.g., for convolutional codes see Conan, 1984; Chang et al., 1997; Katsiotis et al., 2010—to discover encoders that satisfy a criterion. The considered scenario is unique in selecting the code that best fits the data in connecting the IT response to the V1 response. Subsequently, parameters associated with the decoder and the channel can be evaluated or fit to the V4 neural population responses from the same viewed objects. Such analysis would also require initial assumptions about the channel (e.g., continuous vs. discrete) and the decoder prior to performing the iterative searches over their associated parameter spaces. In a different study, Lehky et al. (2011) recorded responses of 674 IT neurons across two monkeys as they were shown 806 objects. The authors analyzed the data in holistic fashion to determine that the heavy tails of the population responses are suggestive of different neurons being tuned to different features. More recently, Dong et al. (2017) incorporated the 806 × 674 data matrix of the aforementioned work to develop simulations for a large number of neuronal responses with various settings for neuron number, stimulus number and identity, and noise level. Through their simulations, the authors justify the findings in Lehky et al. (2011) and also provide an instance of how information can be extracted from a dataset to test additional hypotheses with different assumptions for the underlying processes. Similar to the analysis of Dong et al. (2017), the CTOR hypotheses can be scrutinized by simulating the neuronal responses of the populations in Figures 1, 2 with the variables listed in Appendix D. Although CTOR is a proposition; it is biologically inspired, motivated by prior empirical discussions, and mirrors the tangling-untangling notion that has been accredited within the primate vision community.

## Data Availability Statement

The source code for this study can be found on github (https://github.com/shenghuanjie/ctor).

## Author Contributions

This work was conceived by extensive discussions between all authors. SS and HS conceived the hypothesis and performed the simulations. SS and HS wrote the manuscript with feedback from HP.

## Conflict of Interest

The authors declare that the research was conducted in the absence of any commercial or financial relationships that could be construed as a potential conflict of interest.
