# Supplementary material for: Object Recognition at Higher Regions of the Ventral Visual Stream via Dynamic Inference
Source: Front Comput Neurosci. 2020 Jun 23;14:46. doi: 10.3389/fncom.2020.00046 (PMC7325008; doi:10.3389/fncom.2020.00046)
Supplement: Supplementary file 1 [file Data_Sheet_1.PDF]

Supplementary Material

APPENDIX A

Discussion of the Viterbi algorithm will require several definitions. A prospective **transition** at time k will be defined by the tuple $(\mathbf{b}_{k+1}, \mathbf{b}_k)$. For each prospective transition from time k to $k+1$ a metric

$$\lambda(\mathbf{b}_{k+1}, \mathbf{b}_k) = -\ln(P[\mathbf{b}_{k+1}|\mathbf{b}_k]) - \ln(P[\mathbf{r}_k|\mathbf{b}_{k+1}, \mathbf{b}_k]) \tag{S1}$$

is computed and referred to as the **length** of that prospective transition. It is important to note that the first term on the left-hand-side of (S1) is an a priori probability that is dependent on the stimuli/input, while the second term is a transition probability that depends on the channel. The $\lambda(\cdot, \cdot)$ metric will be computed for each of the possible 2^{L+1} transition pairs. A **path** is defined as a sequence of such transitions over time. For instance, starting at $k = 0$, a path consisting of $K = 4$ transitions would be denoted by $(\mathbf{b}_1, \mathbf{b}_0)$, $(\mathbf{b}_2, \mathbf{b}_1)$, $(\mathbf{b}_3, \mathbf{b}_2)$, $(\mathbf{b}_4, \mathbf{b}_3)$. The Viterbi algorithm also introduces the metrics $\Gamma(\cdot, \cdot)$ and $\Gamma(\cdot)$ via the relation

$$\Gamma(\mathbf{b}_{k+1}, \mathbf{b}_k) = \Gamma(\mathbf{b}_k) + \lambda(\mathbf{b}_{k+1}, \mathbf{b}_k) \tag{S2}$$

which is computed for all $\mathbf{b}_{k+1}, \mathbf{b}_k$ pairs at each k. Accordingly, there will be 2^{L+1} such pairs at each time instant. The computation

$$\Gamma(\mathbf{b}_{k+1}) = \min_{\mathbf{b}_k}\{\Gamma(\mathbf{b}_{k+1}, \mathbf{b}_k)\} \tag{S3}$$

is performed for each $\mathbf{b}_{k+1}$ and the value $\Gamma(\mathbf{b}_{k+1})$ is stored along with the designation $\hat{\mathbf{b}}(b_{k+1}) = \mathbf{b}_k$ for the $\mathbf{b}_k$ that minimizes the above. This $\hat{\mathbf{b}}(b_{k+1})$ is referred to as a **survivor** for time k, and there are 2^L survivors determined at each time instant. An example of this is shown in **Figure S1** where each of the prospective states at time $k+1$ can be reached via a single survivor. The sequence $\hat{\mathbf{b}}(b_0), \hat{\mathbf{b}}(b_1), \ldots, \hat{\mathbf{b}}(b_k)$ is called a survivor path at time k, and the survivor path with the smallest path length corresponds to the MAP recovered sequence. The Viterbi decoding algorithm operates via the following steps:

1) At time $k = 0$ the following initializations are made

$$\begin{aligned} \hat{\mathbf{b}}(b_0) &= \mathbf{b}_0 \\ \Gamma(\mathbf{b}_0) &= 0 \end{aligned} \tag{S4}$$

with $\mathbf{b}_0$ denoting the initial contents (i.e. state) of the encoder.

2) At time $k+1$ compute (S1) for each $\mathbf{b}_{k+1}, \mathbf{b}_k$ pair. For each $\mathbf{b}_{k+1}$ compute and store $\Gamma(\mathbf{b}_{k+1})$ and the corresponding survivor path $\hat{\mathbf{b}}(b_{k+1})$ via the application of (S2) and (S3).

3) Increment k and repeat 2) until $k = K$.

The above steps will terminate at a time K with the smallest length path declared as the survivor $\hat{\mathbf{b}}(b_K)$. By traversing the trellis backwards at time $k = K$ we attain the MAP recovered sequence $\hat{\mathbf{b}}(b_0), \hat{\mathbf{b}}(b_1), \ldots, \hat{\mathbf{b}}(b_K)$.

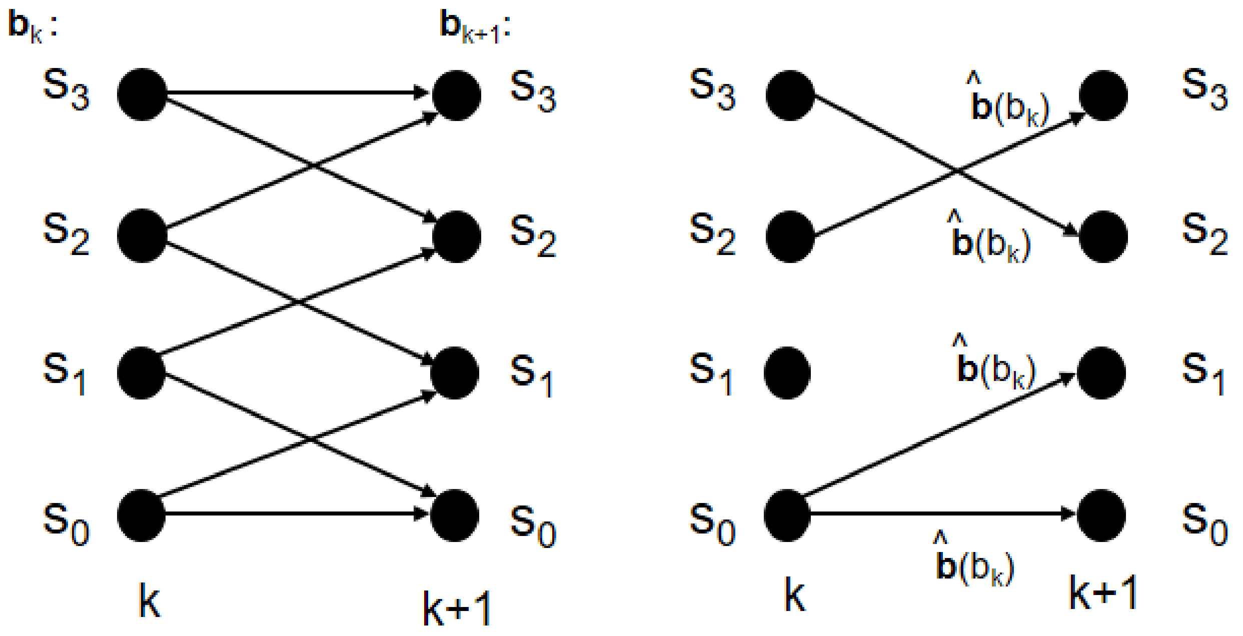

Figure S1. An example of state transitions with $L = 2$ resulting in 4 states and 8 possible transition pairs is shown on the left. The states are indexed via the names S_0, S_1, S_2, and S_3. On the right an example of survival pairs at time k is shown with each $\widehat{\mathbf{b}}(b_k)$ corresponding to a M-tuple that has been selected based on the survival metric of Equation S3.

APPENDIX B

In the decoding process of the simple encoder in **Figure 3**, we can write the computation of transition metrics at all time steps. At time $k = 1$ the decoder computes the transition metrics via (S1) as

$$\begin{aligned}
\lambda(\mathbf{b}_1 = S_0, \mathbf{b}_0 = S_0) &= -\ln(P[DCA|\mathbf{b}_1 = S_0, \mathbf{b}_0 = S_0]) \\
&= -\ln(P[r_1 = D|\text{input} = 0]) - \ln(P[r_1 = C|\text{input} = 0]) \\
&- \ln(P[r_1 = A|\text{input} = 0]) = 4.82 \\
\lambda(\mathbf{b}_1 = S_2, \mathbf{b}_0 = S_0) &= -\ln(P[DCA|\mathbf{b}_1 = S_2, \mathbf{b}_0 = S_0]) \\
&= -\ln(P[r_1 = D|\text{input} = 1]) - \ln(P[r_1 = C|\text{input} = 1]) \\
&- \ln(P[r_1 = A|\text{input} = 1]) = 4.42. \qquad \text{(S5)}
\end{aligned}$$

The two possible transitions above are considered by the Viterbi algorithm because of the encoder's state diagram in **Figure 3**. It should be noted that at $k = 1$ there are two rather than $2^L = 4$ survivors because the decoding has just commenced. Subsequently, the decoder computes

$$\begin{aligned}
\Gamma(\mathbf{b}_1 = S_0, \mathbf{b}_0 = S_0) &= \Gamma(\mathbf{b}_0 = S_0) + \lambda(\mathbf{b}_1 = S_0, \mathbf{b}_0 = S_0) = 0 + 4.82 \\
\Gamma(\mathbf{b}_1 = S_2, \mathbf{b}_0 = S_0) &= \Gamma(\mathbf{b}_0 = S_0) + \lambda(\mathbf{b}_1 = S_2, \mathbf{b}_0 = S_0) = 0 + 4.42. \qquad \text{(S6)}
\end{aligned}$$

At $k = 2$ the decoder considers the prospective transitions

$$\begin{aligned}
\lambda(\mathbf{b}_2 = S_0, \mathbf{b}_1 = S_0) &= -\ln(P[DDB|\mathbf{b}_2 = S_0, \mathbf{b}_1 = S_0]) \\
&= -\ln(P[r_2 = D|\text{input} = 0]) - \ln(P[r_2 = D|\text{input} = 0]) \\
&- \ln(P[r_2 = B|\text{input} = 0]) = 5.809 \\
\lambda(\mathbf{b}_2 = S_2, \mathbf{b}_1 = S_0) &= -\ln(P[DDB|\mathbf{b}_2 = S_2, \mathbf{b}_1 = S_0]) \\
&= -\ln(P[r_2 = D|\text{input} = 1]) - \ln(P[r_2 = D|\text{input} = 1]) \\
&- \ln(P[r_2 = B|\text{input} = 1]) = 3.442 \\
\lambda(\mathbf{b}_2 = S_1, \mathbf{b}_1 = S_2) &= -\ln(P[DDB|\mathbf{b}_2 = S_1, \mathbf{b}_1 = S_2]) \\
&= -\ln(P[r_2 = D|\text{input} = 1]) - \ln(P[r_2 = D|\text{input} = 0]) \\
&- \ln(P[r_2 = B|\text{input} = 1]) = 4.828 \\
\lambda(\mathbf{b}_2 = S_3, \mathbf{b}_1 = S_2) &= -\ln(P[DDB|\mathbf{b}_2 = S_3, \mathbf{b}_1 = S_2]) \\
&= -\ln(P[r_2 = D|\text{input} = 0]) - \ln(P[r_2 = D|\text{input} = 1]) \\
&- \ln(P[r_2 = B|\text{input} = 0]) = 4.422
\end{aligned} \tag{S7}$$

and, as dictated by (S2), computes

$$\begin{aligned}
\Gamma(\mathbf{b}_2 = S_0, \mathbf{b}_1 = S_0) &= \Gamma(\mathbf{b}_1 = S_0) + \lambda(\mathbf{b}_2 = S_0, \mathbf{b}_1 = S_0) = 4.82 + 5.809 = 10.62 \\
\Gamma(\mathbf{b}_2 = S_2, \mathbf{b}_1 = S_0) &= \Gamma(\mathbf{b}_1 = S_0) + \lambda(\mathbf{b}_2 = S_2, \mathbf{b}_1 = S_0) = 4.82 + 3.442 = 8.26 \\
\Gamma(\mathbf{b}_2 = S_1, \mathbf{b}_1 = S_2) &= \Gamma(\mathbf{b}_1 = S_2) + \lambda(\mathbf{b}_2 = S_1, \mathbf{b}_1 = S_2) = 4.42 + 4.828 = 9.24 \\
\Gamma(\mathbf{b}_2 = S_3, \mathbf{b}_1 = S_2) &= \Gamma(\mathbf{b}_1 = S_2) + \lambda(\mathbf{b}_2 = S_3, \mathbf{b}_1 = S_2) = 4.42 + 4.422 = 8.84.
\end{aligned} \tag{S8}$$

At this point in the temporal progression, the above four paths correspond to the $2^L = 4$ survivors since the computation given by (S3) provides

$$\begin{aligned}
\Gamma(\mathbf{b}_2 = S_0) &= 10.62 \\
\Gamma(\mathbf{b}_2 = S_1) &= 9.24 \\
\Gamma(\mathbf{b}_2 = S_2) &= 8.26 \\
\Gamma(\mathbf{b}_2 = S_3) &= 8.84.
\end{aligned} \tag{S9}$$

At $k = 3$ the decoder will consider the transition metrics

$$\begin{aligned}
\lambda(\mathbf{b}_3 = S_0, \mathbf{b}_2 = S_0) &= -\ln(P[DDA|\mathbf{b}_3 = S_0, \mathbf{b}_2 = S_0]) \\
&= -\ln(P[r_3 = D|\text{input} = 0]) - \ln(P[r_3 = D|\text{input} = 0]) \\
&- \ln(P[r_3 = A|\text{input} = 0]) = 5.52 \\
\lambda(\mathbf{b}_3 = S_2, \mathbf{b}_2 = S_0) &= -\ln(P[DDA|\mathbf{b}_3 = S_2, \mathbf{b}_2 = S_0]) \\
&= -\ln(P[r_3 = D|\text{input} = 1]) - \ln(P[r_3 = D|\text{input} = 1]) \\
&- \ln(P[r_3 = A|\text{input} = 1]) = 4.13
\end{aligned}$$

$$
\begin{aligned}
\lambda(\mathbf{b}_3 = S_0, \mathbf{b}_2 = S_1) &= -\ln(P[DDA|\mathbf{b}_3 = S_0, \mathbf{b}_2 = S_1]) \\
&= -\ln(P[r_3 = D|\text{input} = 0]) - \ln(P[r_3 = D|\text{input} = 1]) \\
&- \ln(P[r_3 = A|\text{input} = 1]) = 5.52 \\
\lambda(\mathbf{b}_3 = S_2, \mathbf{b}_2 = S_1) &= -\ln(P[DDA|\mathbf{b}_3 = S_2, \mathbf{b}_2 = S_1]) \\
&= -\ln(P[r_3 = D|\text{input} = 1]) - \ln(P[r_3 = D|\text{input} = 0]) \\
&- \ln(P[r_3 = A|\text{input} = 0]) = 4.13 \\
\lambda(\mathbf{b}_3 = S_1, \mathbf{b}_2 = S_2) &= -\ln(P[DDA|\mathbf{b}_3 = S_1, \mathbf{b}_2 = S_2]) \\
&= -\ln(P[r_3 = D|\text{input} = 1]) - \ln(P[r_3 = D|\text{input} = 0]) \\
&- \ln(P[r_3 = A|\text{input} = 1]) = 5.52 \\
\lambda(\mathbf{b}_3 = S_3, \mathbf{b}_2 = S_2) &= -\ln(P[DDA|\mathbf{b}_3 = S_3, \mathbf{b}_2 = S_2]) \\
&= -\ln(P[r_3 = D|\text{input} = 0]) - \ln(P[r_3 = D|\text{input} = 1]) \\
&- \ln(P[r_3 = A|\text{input} = 0]) = 4.13 \\
\lambda(\mathbf{b}_3 = S_1, \mathbf{b}_2 = S_3) &= -\ln(P[DDA|\mathbf{b}_3 = S_1, \mathbf{b}_2 = S_3]) \\
&= -\ln(P[r_3 = D|\text{input} = 1]) - \ln(P[r_3 = D|\text{input} = 1]) \\
&- \ln(P[r_3 = A|\text{input} = 0]) = 2.74 \\
\lambda(\mathbf{b}_3 = S_3, \mathbf{b}_2 = S_3) &= -\ln(P[DDA|\mathbf{b}_3 = S_3, \mathbf{b}_2 = S_3]) \\
&= -\ln(P[r_3 = D|\text{input} = 0]) - \ln(P[r_3 = D|\text{input} = 0]) \\
&- \ln(P[r_3 = A|\text{input} = 1]) = 6.9
\end{aligned} \tag{S10}
$$

and via (S2) calculate

$$
\begin{aligned}
\Gamma(\mathbf{b}_3 = S_0, \mathbf{b}_2 = S_0) &= \Gamma(\mathbf{b}_2 = S_0) + \lambda(\mathbf{b}_{s3} = S_0, \mathbf{b}_2 = S_0) = 10.62 + 5.52 = 16.14 \\
\Gamma(\mathbf{b}_3 = S_2, \mathbf{b}_2 = S_0) &= \Gamma(\mathbf{b}_2 = S_0) + \lambda(\mathbf{b}_3 = S_2, \mathbf{b}_2 = S_0) = 10.62 + 4.13 = 14.75 \\
\Gamma(\mathbf{b}_3 = S_0, \mathbf{b}_2 = S_1) &= \Gamma(\mathbf{b}_2 = S_1) + \lambda(\mathbf{b}_3 = S_0, \mathbf{b}_2 = S_1) = 9.24 + 5.52 = 14.76 \\
\Gamma(\mathbf{b}_3 = S_2, \mathbf{b}_2 = S_1) &= \Gamma(\mathbf{b}_2 = S_1) + \lambda(\mathbf{b}_3 = S_2, \mathbf{b}_2 = S_1) = 9.24 + 4.13 = 13.37 \\
\Gamma(\mathbf{b}_3 = S_1, \mathbf{b}_2 = S_2) &= \Gamma(\mathbf{b}_2 = S_2) + \lambda(\mathbf{b}_3 = S_1, \mathbf{b}_2 = S_2) = 8.26 + 5.52 = 13.78 \\
\Gamma(\mathbf{b}_3 = S_3, \mathbf{b}_2 = S_2) &= \Gamma(\mathbf{b}_2 = S_2) + \lambda(\mathbf{b}_3 = S_3, \mathbf{b}_2 = S_2) = 8.26 + 4.13 = 12.39 \\
\Gamma(\mathbf{b}_3 = S_1, \mathbf{b}_2 = S_3) &= \Gamma(\mathbf{b}_2 = S_3) + \lambda(\mathbf{b}_3 = S_1, \mathbf{b}_2 = S_3) = 8.84 + 2.74 = 11.58 \\
\Gamma(\mathbf{b}_3 = S_3, \mathbf{b}_2 = S_3) &= \Gamma(\mathbf{b}_2 = S_3) + \lambda(\mathbf{b}_3 = S_3, \mathbf{b}_2 = S_3) = 8.84 + 6.9 = 15.74.
\end{aligned} \tag{S11}
$$

The computation given by (S3) yields the survivor paths

$$
\begin{aligned}
\Gamma(\mathbf{b}_3 = S_0) &= 14.76 \\
\Gamma(\mathbf{b}_3 = S_1) &= 11.58 \\
\Gamma(\mathbf{b}_3 = S_2) &= 13.37 \\
\Gamma(\mathbf{b}_3 = S_3) &= 12.39.
\end{aligned} \tag{S12}
$$

At $k = K = 4$ the calculation of $\{\lambda(\mathbf{b}_4 = S_i, \mathbf{b}_3 = S_j)\}$ is

$$\begin{aligned}
\lambda(\mathbf{b}_4 = S_0, \mathbf{b}_3 = S_0) &= -\ln(P[DDD|\mathbf{b}_3 = S_0, \mathbf{b}_2 = S_0]) \\
&= -\ln(P[r_4 = D|\text{input} = 0]) - \ln(P[r_4 = D|\text{input} = 0]) \\
&- \ln(P[r_4 = D|\text{input} = 0]) = 6.907 \\
\lambda(\mathbf{b}_4 = S_2, \mathbf{b}_3 = S_0) &= -\ln(P[DDD|\mathbf{b}_3 = S_2, \mathbf{b}_2 = S_0]) \\
&= -\ln(P[r_4 = D|\text{input} = 1]) - \ln(P[r_4 = D|\text{input} = 1]) \\
&- \ln(P[r_4 = D|\text{input} = 1]) = 2.748 \\
\lambda(\mathbf{b}_4 = S_0, \mathbf{b}_3 = S_1) &= -\ln(P[DDD|\mathbf{b}_3 = S_0, \mathbf{b}_2 = S_1]) \\
&= -\ln(P[r_4 = D|\text{input} = 0]) - \ln(P[r_4 = D|\text{input} = 1]) \\
&- \ln(P[r_4 = D|\text{input} = 1]) = 4.135 \\
\lambda(\mathbf{b}_4 = S_2, \mathbf{b}_3 = S_1) &= -\ln(P[DDD|\mathbf{b}_3 = S_2, \mathbf{b}_2 = S_1]) \\
&= -\ln(P[r_4 = D|\text{input} = 1]) - \ln(P[r_4 = D|\text{input} = 0]) \\
&- \ln(P[r_4 = D|\text{input} = 0]) = 5.521 \\
\lambda(\mathbf{b}_4 = S_1, \mathbf{b}_3 = S_2) &= -\ln(P[DDD|\mathbf{b}_3 = S_1, \mathbf{b}_2 = S_2]) \\
&= -\ln(P[r_4 = D|\text{input} = 1]) - \ln(P[r_4 = D|\text{input} = 0]) \\
&- \ln(P[r_4 = D|\text{input} = 1]) = 4.135 \\
\lambda(\mathbf{b}_4 = S_3, \mathbf{b}_3 = S_2) &= -\ln(P[DDD|\mathbf{b}_3 = S_3, \mathbf{b}_2 = S_2]) \\
&= -\ln(P[r_4 = D|\text{input} = 0]) - \ln(P[r_4 = D|\text{input} = 1]) \\
&- \ln(P[r_4 = D|\text{input} = 0]) = 5.521 \\
\lambda(\mathbf{b}_4 = S_1, \mathbf{b}_3 = S_3) &= -\ln(P[DDD|\mathbf{b}_3 = S_1, \mathbf{b}_2 = S_3]) \\
&= -\ln(P[r_4 = D|\text{input} = 1]) - \ln(P[r_4 = D|\text{input} = 1]) \\
&- \ln(P[r_4 = D|\text{input} = 0]) = 4.135 \\
\lambda(\mathbf{b}_4 = S_3, \mathbf{b}_3 = S_3) &= -\ln(P[DDD|\mathbf{b}_3 = S_3, \mathbf{b}_2 = S_3]) \\
&= -\ln(P[r_4 = D|\text{input} = 0]) - \ln(P[r_4 = D|\text{input} = 0]) \\
&- \ln(P[r_4 = D|\text{input} = 1]) = 5.521.
\end{aligned} \tag{S13}$$

and the resultant path metrics

$$\begin{aligned}
\Gamma(\mathbf{b}_4 = S_0, \mathbf{b}_3 = S_0) &= \Gamma(\mathbf{b}_3 = S_0) + \lambda(\mathbf{b}_4 = S_0, \mathbf{b}_3 = S_0) = 14.76 + 6.907 = 21.66 \\
\Gamma(\mathbf{b}_4 = S_2, \mathbf{b}_3 = S_0) &= \Gamma(\mathbf{b}_3 = S_0) + \lambda(\mathbf{b}_4 = S_2, \mathbf{b}_3 = S_0) = 14.76 + 2.748 = 17.5 \\
\Gamma(\mathbf{b}_4 = S_0, \mathbf{b}_3 = S_1) &= \Gamma(\mathbf{b}_3 = S_1) + \lambda(\mathbf{b}_4 = S_0, \mathbf{b}_3 = S_1) = 11.58 + 4.135 = 15.71 \\
\Gamma(\mathbf{b}_4 = S_2, \mathbf{b}_3 = S_1) &= \Gamma(\mathbf{b}_3 = S_1) + \lambda(\mathbf{b}_4 = S_2, \mathbf{b}_3 = S_1) = 11.58 + 5.521 = 17.1 \\
\Gamma(\mathbf{b}_4 = S_1, \mathbf{b}_3 = S_2) &= \Gamma(\mathbf{b}_3 = S_2) + \lambda(\mathbf{b}_4 = S_1, \mathbf{b}_3 = S_2) = 13.37 + 4.135 = 17.5 \\
\Gamma(\mathbf{b}_4 = S_3, \mathbf{b}_3 = S_2) &= \Gamma(\mathbf{b}_3 = S_2) + \lambda(\mathbf{b}_4 = S_3, \mathbf{b}_3 = S_2) = 13.37 + 5.521 = 18.89
\end{aligned}$$

$$
\begin{aligned}
\Gamma(\mathbf{b}_4 = S_1, \mathbf{b}_3 = S_3) &= \Gamma(\mathbf{b}_3 = S_3) + \lambda(\mathbf{b}_4 = S_1, \mathbf{b}_3 = S_3) = 12.39 + 4.135 = 16.52 \\
\Gamma(\mathbf{b}_4 = S_3, \mathbf{b}_3 = S_3) &= \Gamma(\mathbf{b}_3 = S_3) + \lambda(\mathbf{b}_4 = S_3, \mathbf{b}_3 = S_3) = 12.39 + 5.521 = 17.91
\end{aligned} \tag{S14}
$$

along with the computation in (S3) provide the survivor paths

$$
\begin{aligned}
\Gamma(\mathbf{b}_4 = S_0) &= 15.71 \\
\Gamma(\mathbf{b}_4 = S_1) &= 16.52 \\
\Gamma(\mathbf{b}_4 = S_2) &= 17.1 \\
\Gamma(\mathbf{b}_4 = S_3) &= 17.91.
\end{aligned} \tag{S15}
$$

APPENDIX C

For the decoder to compute and update the a priori probabilities $P[\mathbf{b}_{k+1}|\mathbf{b}_k]$, it must have knowledge of encoder structure. The simple encoder in **Figure 3** has four possible states: $S_0 = 00$, $S_1 = 10$, $S_2 = 01$, $S_3 = 11$. For the sequence $\tilde{\mathbf{b}}^c = 1100\ 1110$, that has been compressed and committed to memory, the a priori probabilities are calculated by counting the possible state transitions. When considering $\tilde{\mathbf{b}}^c$ for $k = 0, 1, \ldots, 8$ we have the sequence of states: $\mathbf{b}_0 = S_0$, $\mathbf{b}_1 = S_2$, $\mathbf{b}_2 = S_3$, $\mathbf{b}_3 = S_1$, $\mathbf{b}_4 = S_0$, $\mathbf{b}_5 = S_2$, $\mathbf{b}_6 = S_3$, $\mathbf{b}_7 = S_3$, $\mathbf{b}_8 = S_1$, from which it is possible to estimate the likelihood of a subsequent state given a current state. For instance, to calculate $P[\mathbf{b}_{k+1} = S_1|\mathbf{b}_k = S_3]$, it is noted that $\mathbf{b}_k = S_3$ occurs three times (i.e. $k = 2, 6, 7$), and that the number of occurrences of $\mathbf{b}_{k+1} = S_1|\mathbf{b}_k = S_3$ is 2 (for $k = 2, 7$). Thus, an estimate for the a priori probability $P[\mathbf{b}_{k+1} = S_1|\mathbf{b}_k = S_3]$ is $2/3$. Following this procedure, estimates for all a priori probabilities are listed as:

$$
\begin{aligned}
P[\mathbf{b}_{k+1} = S_0|\mathbf{b}_k = S_0] &= 0 \\
P[\mathbf{b}_{k+1} = S_1|\mathbf{b}_k = S_0] &= 0 \\
P[\mathbf{b}_{k+1} = S_2|\mathbf{b}_k = S_0] &= 1 \\
P[\mathbf{b}_{k+1} = S_3|\mathbf{b}_k = S_0] &= 0 \\
P[\mathbf{b}_{k+1} = S_0|\mathbf{b}_k = S_1] &= 1 \\
P[\mathbf{b}_{k+1} = S_1|\mathbf{b}_k = S_1] &= 0 \\
P[\mathbf{b}_{k+1} = S_2|\mathbf{b}_k = S_1] &= 0 \\
P[\mathbf{b}_{k+1} = S_3|\mathbf{b}_k = S_1] &= 0 \\
P[\mathbf{b}_{k+1} = S_0|\mathbf{b}_k = S_2] &= 0 \\
P[\mathbf{b}_{k+1} = S_1|\mathbf{b}_k = S_2] &= 0 \\
P[\mathbf{b}_{k+1} = S_2|\mathbf{b}_k = S_2] &= 0 \\
P[\mathbf{b}_{k+1} = S_3|\mathbf{b}_k = S_2] &= 1 \\
P[\mathbf{b}_{k+1} = S_0|\mathbf{b}_k = S_3] &= 0 \\
P[\mathbf{b}_{k+1} = S_1|\mathbf{b}_k = S_3] &= 2/3 \\
P[\mathbf{b}_{k+1} = S_2|\mathbf{b}_k = S_3] &= 0
\end{aligned}
$$

$$P[\mathbf{b}_{k+1} = S_3 | \mathbf{b}_k = S_3] \quad = \quad 1/3. \tag{S16}$$

APPENDIX D

A table of symbols used throughout the manuscript and their corresponding definitions.

Symbol	Computational Description	Biological Description
K	Number of time epochs considered in to convey a message.	The number of time epochs necessary to form the representation that the viewed object should evoke at the IT.
M	Number of information-bearing bits processed by the encoder at each time epoch.	The number of bits processed by the retinal, LGN, and V1 circuitry during a particular epoch.
N	The length of a codeword generated by the encoder per M input bits at each time epoch.	Dimensionality increase applied to the viewed representation by the retinal, LGN, and V1 circuitry.
L	Encoder constraint length, or – in the simplest scenario – the total number of shift registers in an encoder.	A measure of the memory that a portion (i.e. bit) of the viewed representation has on the lower-level encoding of the ensuing portions of the representation.
$\mathbf{b}_k^i$	The M-bit encoder input at time k. The index i denotes the object identity.	The neural response that should be evoked by the IT when presented with viewed object with identity i.
$\mathbf{s}_k$	The encoder output following the viewing of an object at time k.	The output of the retina, LGN, and V1 stages when viewing object at time k.
$\mathbf{r}$	The channel output.	The tangled and noisy neural response of the viewed object presented to the IT.
$P[\mathbf{r}\|\mathbf{s}]$	The conditional distribution modeling the channel that separates the encoder from the decoder.	The perturbation applied to the viewed object via neural noise along the VVS as well as by the environment during the viewing of the object.
$\mathbf{n}$	The noise process in the case of an additive, continuous channel.	A perturbation applied to the representation of the viewed object via the viewing environment and the neural noise.
$\mathbf{w}$	A weight vector of length KM applied to the decoder output to recover the category of the viewed object.	The combining of the untangled signal by the neurons in the IT to arrive at a category for the viewed object.

$S_1, S_2, \ldots, S_{2L}$	The possible states that the encoder can take at a given time.	A state of the neural populations of the lower visual areas while tangling the representation that the viewed object should evoke at the IT for proper recognition. This will affect how future inputs are tangled.
$P[\mathbf{r}_k \| \mathbf{b}_{k+1}, \mathbf{b}_k]$	The likelihood of the state transition $\mathbf{b}_k$ to $\mathbf{b}_{k+1}$ having led to the sequence $\mathbf{r}$ at time $k+1$.	The likelihood of a tangled representation having arisen at the present time in light of what was viewed in the time epoch before.
$P[\mathbf{b}_{k+1} \| \mathbf{b}_k]$	The a priori probability of expecting one state in the next time instant after having viewed a state at the current time k.	A belief provided by memory to IT about what will be viewed in the subsequent time instant in light of what is currently viewed.
$\lambda(\mathbf{b}_{k+1} = S_i, \mathbf{b}_k = S_j)$	The length of a prospective state transition during the decoding operation.	A measure of the IT neurons' response towards the viewed object matching a representation in memory.
$\Gamma(\mathbf{b}_{k+1} = S_i, \mathbf{b}_k = S_j)$	The aggregate length of a state transitions constituting a path through a trellis during the decoding process.	The cumulative response of IT neurons to viewed object leading to a declaration of the representation that was viewed.
$\widehat{\mathbf{b}},\ \widehat{\mathbf{b}}(b_k)$	The recovered sequence by the decoder.	The reconstructed object identity at the IT based on the tangled, perturbed representation that the IT has received from the lower visual layers and its a priori belief.
F	The number of features comprising the viewed object.	The dimensionality of features determined as being important for the viewed object by the VVS pathway.
$\widetilde{\mathbf{b}} : {}^{i}\widehat{b}_j$ $i = 1, 2, \ldots, F$ $j = 1, 2, \ldots, KM$	The interleaved version of the decoded output.	An ordering of the features of the object representation at the IT according to their perceived importance.
$\widetilde{\mathbf{b}}^c$	The compressed version of the decoded output.	The compressed representation of viewed objects in memory circuitry of hippocampus and amygdala.
